# The Burden Attributable to Mental and Substance Use Disorders as Risk Factors for Suicide: Findings from the Global Burden of Disease Study 2010

**DOI:** 10.1371/journal.pone.0091936

**Published:** 2014-04-02

**Authors:** Alize J. Ferrari, Rosana E. Norman, Greg Freedman, Amanda J. Baxter, Jane E. Pirkis, Meredith G. Harris, Andrew Page, Emily Carnahan, Louisa Degenhardt, Theo Vos, Harvey A. Whiteford

**Affiliations:** 1 University of Queensland, School of Population Health, Herston, Queensland, Australia; 2 Queensland Centre for Mental Health Research, Wacol, Queensland, Australia; 3 Queensland Children's Medical Research Institute, University of Queensland, Herston, Queensland, Australia; 4 Institute for Health Metrics and Evaluation, University of Washington, Seattle, Washington, United States of America; 5 Melbourne School of Population and Global Health, University of Melbourne, Melbourne, Australia; 6 School of Science and Health, University of Western Sydney, Campbelltown, New South Wales, Australia; 7 National Drug and Alcohol Research Centre, University of New South Wales, Sydney, Australia; University of Adelaide, Australia

## Abstract

**Background:**

The Global Burden of Disease Study 2010 (GBD 2010) identified mental and substance use disorders as the 5^th^ leading contributor of burden in 2010, measured by disability adjusted life years (DALYs). This estimate was incomplete as it excluded burden resulting from the increased risk of suicide captured elsewhere in GBD 2010's mutually exclusive list of diseases and injuries. Here, we estimate suicide DALYs attributable to mental and substance use disorders.

**Methods:**

Relative-risk estimates of suicide due to mental and substance use disorders and the global prevalence of each disorder were used to estimate population attributable fractions. These were adjusted for global differences in the proportion of suicide due to mental and substance use disorders compared to other causes then multiplied by suicide DALYs reported in GBD 2010 to estimate attributable DALYs (with 95% uncertainty).

**Results:**

Mental and substance use disorders were responsible for 22.5 million (14.8–29.8 million) of the 36.2 million (26.5–44.3 million) DALYs allocated to suicide in 2010. Depression was responsible for the largest proportion of suicide DALYs (46.1% (28.0%–60.8%)) and anorexia nervosa the lowest (0.2% (0.02%–0.5%)). DALYs occurred throughout the lifespan, with the largest proportion found in Eastern Europe and Asia, and males aged 20–30 years. The inclusion of attributable suicide DALYs would have increased the overall burden of mental and substance use disorders (assigned to them in GBD 2010 as a direct cause) from 7.4% (6.2%–8.6%) to 8.3% (7.1%–9.6%) of global DALYs, and would have changed the global ranking from 5^th^ to 3^rd^ leading cause of burden.

**Conclusions:**

Capturing the suicide burden attributable to mental and substance use disorders allows for more accurate estimates of burden. More consideration needs to be given to interventions targeted to populations with, or at risk for, mental and substance use disorders as an effective strategy for suicide prevention.

## Introduction

There has been growing recognition of the importance of mental and substance use disorders as contributors to health loss in all countries. The Global Burden of Disease Study 2010 (GBD 2010) is the largest and most recent effort to quantify this by systematically integrating years of life lost (YLLs) and years of life with disability (YLDs) into disability adjusted life years (DALYs) for diseases, injuries and risk factors [1]–[7].

GBD 2010 presented age-, sex-, year-, country-, and region-specific DALYs for 291 diseases and injuries as well as for 67 risk factors [1]–[7]; using improved methodology compared to previous GBD studies [8], [9]. Mental and substance use disorders explained 7.4% (95% uncertainty interval: 6.2–8.6%) of total DALYs in 2010, confirming them as the leading disease category of YLDs, and the 5^th^ leading category of DALYs globally [10]–[12]. This estimate reflects ‘direct burden’ where mental and substance use disorders are the direct cause of health loss, but excludes the excess (attributable) burden resulting from the increased risk of mortality and disability due to subsequent health outcomes captured elsewhere in the mutually exclusive disease and injury categories in GBD 2010. Jointly considering the direct and the attributable burden of mental and substance use disorders provides an estimation of the putative causal relationship between the disorders and other health outcomes. This is of clinical and policy relevance as it clearly delineates the disability and mortality that potentially can be modified by interventions to prevent and treat mental and substance use disorders.

Here, we expand on the published GBD 2010 findings by estimating the additional burden attributable to mental and substance use disorders as risk factors for suicide. Suicide, defined as deaths caused by intentional, self-inflicted poisoning or injury [13], was the 13^th^ leading cause of YLLs worldwide in 2010 [1], [6]. Nearly 1 million people complete suicide every year with over 50% aged between 15 and 44 years [14], [15]. Over 80% of suicides occur in low to middle income countries and close to 50% occur in India and China alone [15], [16]. Suicide from firearms, car exhaust and poisoning are more common in high income countries and suicide from pesticide poisoning, hanging and self-immolation are more common in low to middle income countries [17]. It is important to consider these differences in the global epidemiology of suicide while quantifying the suicide burden attributable to mental and substance use disorders.

The link between mental and substance use disorders and suicide is well documented [14]–[20] and authors such as Prince and colleagues argued [14] that failure to include suicide as part of mental and substance use disorder estimates in the previous GBD studies [8], [9] led to an underestimate of the extent of the burden. A literature review and meta-analysis by Harris and Barraclough showed that of the 249 studies and 44 mental disorders assessed, 36 disorders were associated with an increased risk of suicide [19]. Li and collaborators also found that the risk of suicide was 7.5 (6.2–9.0) times higher in males and 11.7 (9.7–14.1) times higher in females with a mental or substance use disorder compared to males and females with no disorder. Depression and bipolar disorder accounted for the highest risk [20]. Even when other risk factors such as adverse marital effects, employment and socio-economic status were considered, mental and substance use disorders remain strongly associated with suicide [20], [21].

Quantifying the suicide burden attributable to mental and substance use disorders also corrects for the low burden from premature mortality (YLLs) directly attributed to mental and substance use disorders in GBD 2010. Although mental and substance use disorders were identified as a leading cause of global burden, YLDs contributed to 95% of DALYs [5], [10]. In spite of evidence of excess mortality attributable to many mental and substance use disorders, only substance use disorders, anorexia nervosa, and schizophrenia are recognized as underlying causes of death in the International Classification of Diseases (ICD-10) cause of death guidelines [13] used in GBD 2010. Even for those disorders, few deaths were captured in the vital registrations used in the estimation of YLLs, as this typically involves the cumbersome task of disentangling the effect of multiple mental, substance and physical disorders to identify primary cause of death.

Investigating mental and substance use disorders as risk factors for fatal outcomes like suicide allows us to circumvent this problem by making use of GBD 2010's comparative risk analysis (CRA) methodology [7]. Rather than rely on certification and coding practices in mortality registration systems, this method allows quantification of the difference in population health in a counterfactual with a theoretical minimum level of exposure [7]. We make use of this method here to calculate the suicide burden attributable to mental and substance use disorders, and examine variations by region, country, age, year and disorder.

## Methods

The suicide burden attributable to mental and substance use disorders was estimated by comparing the current health status with a theoretical-minimum-risk exposure defined as the counterfactual status of the absence of mental and substance use disorders. Population attributable fractions (PAFs) were determined from the prevalence of exposure to each disorder and the relative-risk (RR) of suicide [7]. For each disorder this involved:

1. Reviewing the strength of the evidence for a causal relationship between the disorder and suicide.
2. Expanding on existing systematic reviews of the literature quantifying the effect size for the disorder as a risk factor for suicide. The preferred metric was population-representative RR estimates.
3. Pooling all RR estimates using meta-analysis.
4. Combining the pooled RR estimate with GBD 2010 prevalence estimates to generate PAFs by age, sex, country, and year.
5. Adjusting PAFs for global differences in suicide attributable to mental and substance use disorders versus differences attributable to other causes.
6. Multiplying PAFs by suicide YLLs reported in GBD 2010 to estimate attributable burden.

### Case definition

GBD methods suggest that for each risk factor-outcome pairing, there should be (1) sufficient data to enable estimation of relative effect sizes as well as (2) sufficient evidence for a causal effect [7]. A literature review by Baxter and collaborators [22] as well as other studies summarised in the previous section [14]–[20] investigating mental and substance use disorders as risk factors for other health outcomes found sufficient evidence to meet these two conditions for suicide.

Mental and substance use disorders investigated were those included in GBD 2010 for which there was evidence of an increased risk of suicide [10], [19], [20]. These were major depressive disorder (MDD), bipolar disorder, schizophrenia, anxiety disorder, anorexia nervosa, alcohol dependence, amphetamine dependence, cocaine dependence and opioid dependence. All disorders were defined using the Diagnostic and Statistical Manual of Mental disorders (DSM) [23] or ICD diagnostic criteria [13]. Suicide was defined as cases meeting ICD-10 cause of death codes for intentional self-inflicted poisoning or injury (X60–X84) [13]. In some countries a large proportion of injury-related deaths are coded as ‘underdetermined intent’ for cultural, religious or medico-legal reasons. GBD 2010 developed a method to redistribute these deaths to specific underlying causes, including suicide [6]. Although GBD 2010 also considered the effects of attempted suicide as ‘non-fatal self-harm’ [5], this was not investigated in this paper.

### Literature search to identify relative-risk estimates

We used data sources from recent and methodologically comparable systematic reviews of the association between suicide and mental and substance use disorders [20], [24]–[27], specifically affective disorders, anxiety disorders, schizophrenia (14 studies from these 3 disorder groups) [20], cocaine, opioid, and amphetamine dependence (24 studies) [24]–[26] and alcohol dependence (12 studies) [27]. We expanded the Li and collaborators systematic review and replicated the literature search [20] to collect data for bipolar disorder and MDD separately (rather than affective disorders combined), and anorexia nervosa which was not included in the original review. The search strategy used was in keeping with the Preferred Reporting Items for Systematic Reviews and Meta-analyses (PRISMA) statement [28] (See Text S1 in File S1 for the PRISMA checklist and flow diagram). Electronic databases (Medline and Embase) were searched between 1966 and 2010. A secondary search of reference lists and the grey literature was also conducted. Studies were included that; (1) considered mental and substance use disorders as a risk factor associated with suicide; (2) reported a RR with 95% uncertainty, or provided sufficient information for these to be calculated; (3) were individual-level case-control or cohort studies where a clear temporal association between exposure and outcome could be determined; (4) had a minimum follow up period of 1 year and; (5) included disorders based on ICD [13] or DSM [23] nomenclature to ensure consistency in case definitions. Sex-specific data were preferred but non sex-specific estimates were included (e.g. for substance use disorders) where data were sparse. For each study, information on study methodology, quality and findings were extracted into a Microsoft Excel spreadsheet. See Table S1 in File S1 for a summary of the study variables extracted.

### Meta-analysis of relative-risk estimates

For each disorder (except alcohol dependence for which a pooled estimate was available [27]), MetaXL software, an add-in for Microsoft Excel [29], was used to pool RR estimates from different studies. This was done for males and females separately and also combined. RR estimates were pooled using a random effects model, and if there was sufficient data to do so, a quality effects model [30]. Pooled RRs from the quality-effects model were preferred as these gave greater weight to studies of high quality versus studies of lesser quality, and avoided the anomaly of random effects models which revert to equal weighting regardless of sample size if heterogeneity is large [30]–[32]. Study quality was assessed using a quality index which scored studies based on sampling design and representativeness and also the availability of age- and gender-specific estimates. It was limited to these items to reduce potential subjectivity within and between quality scores. To prevent inter-rater bias, all studies were rated by one researcher and a random sample of scores was checked by an independent researcher. See Table S1 in File S1 for the quality index and scores.

### Prevalence of mental and substance use disorders

We obtained the prevalence distribution of each mental and substance use disorder from the epidemiological disease models used in the calculation of direct burden (i.e. YLDs) in GBD 2010 [10], [12]. These were based on a separate literature review (presented in greater detail elsewhere [33]–[38]) conducted between 1980 to 2010 to capture studies reporting prevalence, incidence, remission, duration and all cause-excess mortality associated with mental and substance use disorders. Point (current or past month) prevalence estimates of DSM/ICD defined disorders were required. Twelve-month prevalence estimates were accepted to maximize inclusion but adjusted towards the level of point prevalence using study-level covariates. Lifetime prevalence was excluded as it is more likely than point or period prevalence to be affected by recall bias [39], [40]. GBD 2010's DisMod-MR, a Bayesian meta-regression tool, was used to integrate these estimates into an epidemiological disease model. From the epidemiological inputs, DisMod-MR generated prevalence by sex and age for 187 countries, 21 world regions and 1990, 2005 and 2010 [2], [41]. Prevalent cases for each disorder have been summarised in previous publications [2], [10], [12].

### Population attributable fractions

PAFs were calculated from the DisMod-MR prevalence output (P) for each disorder and the pooled RR of suicide given exposure to the disorder. PAFs were calculated by age, sex, country, year and disorder (consistent with the format of GBD 2010 estimates) using the following formula [42]:
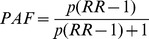
Given the presence of comorbidity between mental and substance use disorders, disorder-specific PAFs cannot be summed to obtain the ‘joint effect’ of combined mental and substance use disorders on suicide. Instead, a joint PAF was estimated using the multiplicative method of adjusting for comorbidity between disorders [43]. This can be understood as calculating the complement of the product of the complements of each individual PAF. The following formula was used where *i* is the individual risk factor, and *n* is the total number of risk factors [7];
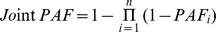



### Ceiling values for joint population attributable fractions

Although studies from high income countries have consistently shown that up to 90% of suicides occur as a result of an underlying mental or substance use disorder [18], [21], [44], there is also evidence to suggest that this proportion is substantially lower in China, Taiwan and India; where symptoms of ‘dysphoric affect’ and ‘impulsivity’ (which do not constitute a mental and substance use disorder) are expressed through more lethal methods of self-harming such as pesticide poisoning and self-immolation [45]–[48]. This in turn, increases the number of completed suicides occurring from self-harm behaviours (characteristically instigated as impulsive acts, without the presence of a mental and substance use disorder or a clear intent to die) in these countries which would have resulted in an “attempted suicide” had such methods not been available [46], [47].

So as not to overestimate the total proportion of suicide burden attributable to mental and substance use disorders, we first portioned out global differences in suicide attributable to mental and substance use disorders from differences attributable to other causes. More specifically, the total proportion of suicide cases attributable to mental and substance use disorders in different countries was calculated and used to set a ceiling value (or upper threshold) for the joint PAFs. We examined reference lists of existing reviews for psychological autopsy studies [18], [21], [44] and conducted a supplementary literature search to capture additional data sources up to 2010. The psychological autopsy method is a retrospective assessment of causes of death which involves canvassing the views of individuals closest to the deceased and substantiating evidence from sources such as hospital and police records [49]. The overall number of suicide cases attributable to mental and substance use disorders was extracted from these studies if DSM/ICD diagnostic criteria [13], [23] were used and the number of attributable suicide cases was reported for mental and substance use disorders as a group rather than for individual disorders. If gender was not recorded we also accepted combined estimates for males and females. Given that there were insufficient data to calculate ceiling values individually for each country or region, we pooled estimates into 2 broad categories based on the percentage of suicide cases reported to be due to mental and substance use disorders. Meta-analyses based on quality effects models were used to generate separate pooled proportions for Group 1: China, India and Taiwan and Group 2: all other countries.

These calculated proportions of suicide cases due to mental and substance use disorder were used to set the ceiling value of joint PAFs. All quantities of interest in GBD 2010 were calculated a thousand times in order to incorporate all sources of uncertainty. Similarly, we created a thousand draws of the ‘ceiling values’ based on the pooled estimates of mean and standard error. When estimating the joint PAFs of suicide attributed to all mental and substance use disorders we did not allow PAF estimates in any of the one thousand draws to exceed the ceiling value in the corresponding draw. For draws that did exceed the ceiling, we scaled down each of the component mental and substance use disorder PAFs by the ratio of the ceiling to the combined PAF.

### Attributable burden

The final step was to multiply PAFs by the corresponding GBD 2010 YLLs for suicide [5], [6] to calculate attributable burden. Since only completed suicides were considered in our analyses, only YLLs were included in attributable DALY estimates. To quantify 95% uncertainty around our final burden estimate we calculated attributable YLLs and DALYs at the one thousand draw level and bounded the 95% uncertainty interval by the 2.5 and 97.5 centile values. All reporting of DALYs by region and country is based on age-standardised estimates using direct standardization to the global standard population proposed by the World Health Organization (WHO) in 2001 [50].

## Results

### Pooled relative-risk estimates

Our search culminated in a dataset of 40 studies and 85 RR estimates covering 14 countries (Table S1 in File S1 summarizes included studies). There was a statistically significant increased risk of suicide for all selected mental and substance use disorders (table 1). The greatest risk was seen in MDD followed by schizophrenia, and alcohol dependence. The 95% confidence intervals around each pooled RR indicated high levels of uncertainty with statistical heterogeneity (as measured by the I^2^ statistic) of up to 90%. A statistically significant sex difference was only observed for alcohol dependence (Table S2 in File S1 summarizes sex-specific pooled RRs) hence the overall pooled proportions for both sexes combined were used in PAF calculations. Given that the one RR estimate for amphetamine dependence was not statistically different (i.e. occurred within overlapping 95% uncertainty) to the three estimates for cocaine dependence, we combined them to calculate a pooled RR for all psychostimulants. This was used to calculate PAFs for both disorders.

**Table 1:** Pooled relative-risk of suicide in those diagnosed with a mental or substance use disorder.

Disorder	Number of studies	Pooled relative risk (95% UI)
Major depressive disorder	4	19.9 (9.5–41.7)
Anxiety disorder	7	2.7 (1.7–4.3)
Schizophrenia	4	12.6 (11.0–14.5)
Bipolar disorder	4	5.7 (2.6–12.4)
Anorexia nervosa	9	7.6 (2.2–25.6)
Alcohol dependence^b^	12	9.8 (9.0–10.7)
Opioid dependence	21	6.9 (4.5–10.5)
Psychostimulant dependence	4	8.2 (3.9–16.9)
Amphetamine dependence^a^	1	4.5(1.1–9.03)
Cocaine dependence^a^	3	16.9(6.01–47.2)

*Note. 95% UI: 95% uncertainty interval;*

a *Due to lack of data, simultaneously pooled cocaine and amphetamine relative-risk estimates into an overall estimate for psychostimulants which was applied to both disorders;*

b *Used reported pooled standardised mortality ratios from Wilcox et al* [*27*] *for alcohol dependence.*

### Ceiling values for joint PAFs

Out of 166 psychological autopsy studies reviewed, 43 studies and 57 estimates covering 20 countries were used to calculate ceiling value for joint PAFs (Table S3 in File S1 summarizes included studies). In China, India and Taiwan (group 1), 68.3% (55.2%–80.0%) of suicide cases was due to mental and substance use disorders which was lower than in all other countries (group 2), where 84.5% (78.6%–89.6%) of suicide cases were due to mental and substance use disorders. These two pooled proportions were used as the ceiling values for joint PAFs from China, India and Taiwan (Group 1) and all other countries (Group 2) respectively. Note that there was considerable heterogeneity between studies. As we found no statistically significant sex difference, the overall pooled proportions were used in PAF calculations (Table S4 in File S1 summarizes sex-specific pooled proportions).

### Attributable burden

Mental and substance use disorders were responsible for 22.5 million (14.8–29.8 million) of the 36.2 million (26.5–44.3 million) DALYs allocated to suicide in 2010, amounting to 62.1% (43.8%–75.3%) of total suicide DALYs. The proportion of attributable suicide DALYs in 1990 was almost identical to that in 2010 (62.1% (44.5%–75.4%)). The remainder of this section focuses on 2010 estimates with 1990 estimates summarised in Table S5 in File S1.

**Figure 1:**
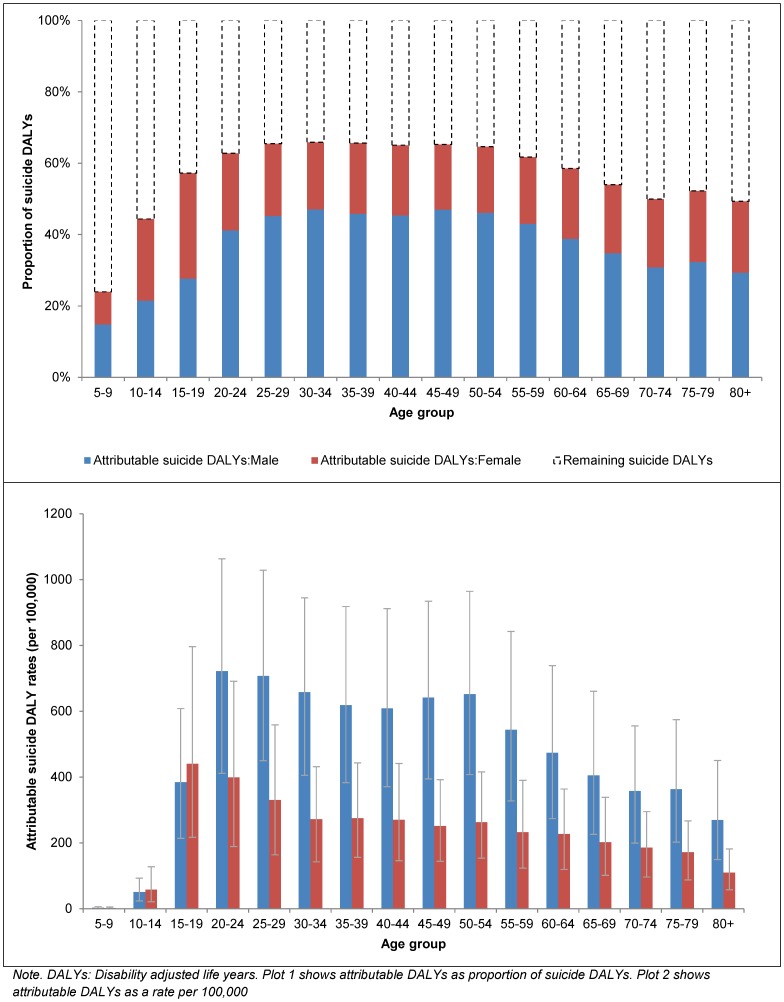
Suicide DALYs attributable to mental and substance use disorders by age and sex, in 2010.

There were twice as many mental and substance use disorders attributable suicide DALYs for males (14.9 million (9.5–20.1 million)) compared to females (7.6 million (4.4–10.6 million)). For all disorders, this sex difference was consistent throughout the lifespan. Attributable suicide DALYs were apparent from those aged ≥5 years, with the highest proportion occurring between those aged 20–30 years (Figure 1).

**Figure 2:**
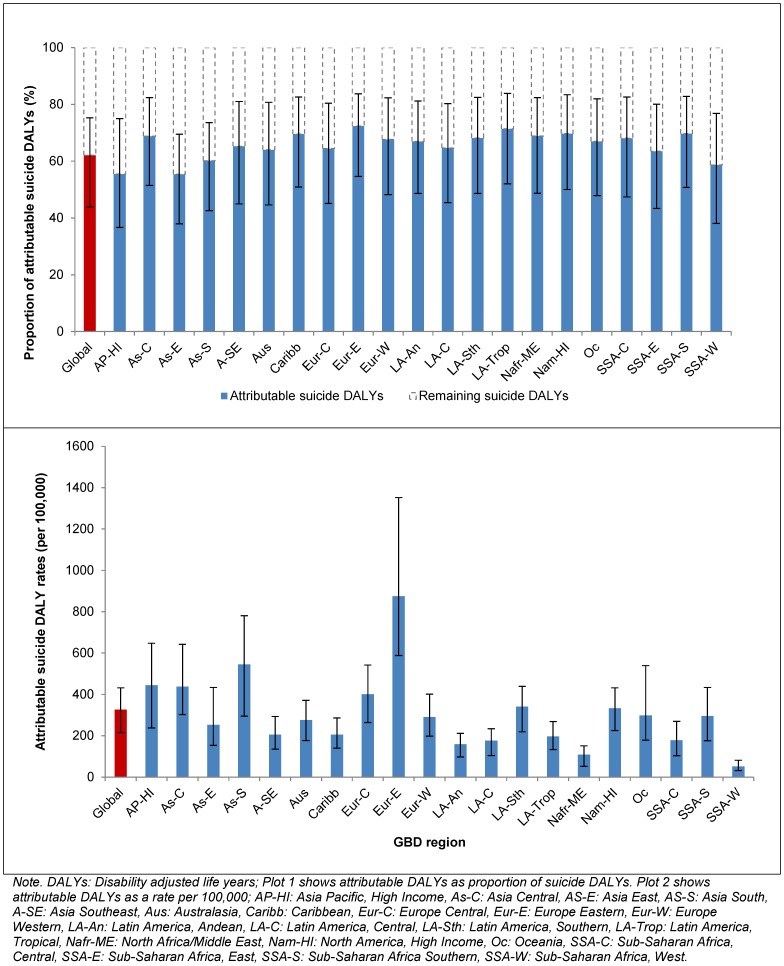
Suicide DALYs attributable to mental and substance use disorders by region, in 2010.

The proportion of suicide DALYs explained by mental and substance use disorders was reasonably consistent between regions and within the range of the ceiling values presented in the previous section. When considered in terms of absolute DALYs, Asia South and Asia East had the highest burden attributable to mental and substance use disorders, given their large population size. In terms of age-standardized rates, Europe Eastern had the highest burden (almost 3 times higher than the global mean) and Sub-Saharan Africa West the lowest (6 times lower than the global mean) (Figure 2 and Table S5 in File S1 summarize attributable DALYs by disorder, region, age and sex).

**Figure 3:**
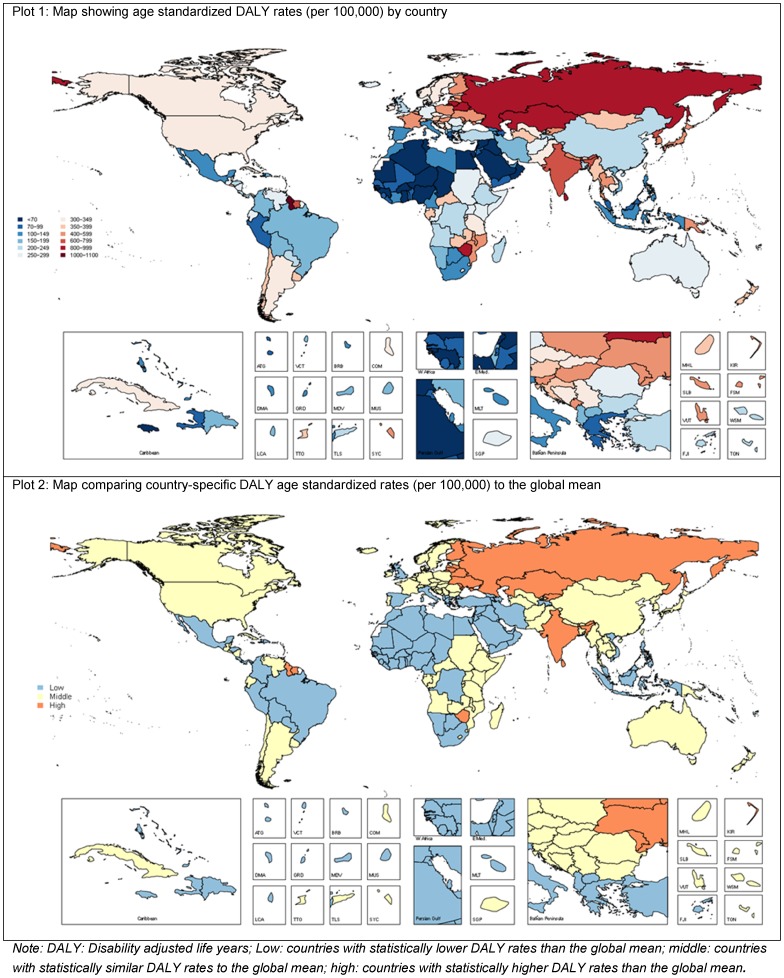
Suicide DALYs (rates per 100,000) attributable to mental and substance use disorders by country, in 2010.

There were also differences in attributable suicide DALYs across countries (plot 1, figure 3). Attributable DALY rates were highest in Kazakhstan and lowest in Saudi Arabia, however many of the country level differences presented in plot 1 were within overlapping ranges of uncertainty (plot 2, figure 3). Except for Guyana, Suriname and Zimbabwe, all countries with statistically higher attributable DALY rates than the global mean were from Eastern Europe and South Asia. Countries with statistically lower DALY rates than the global mean included those from South America, Oceania, Africa and the Middle East and parts of Asia.

**Figure 4:**
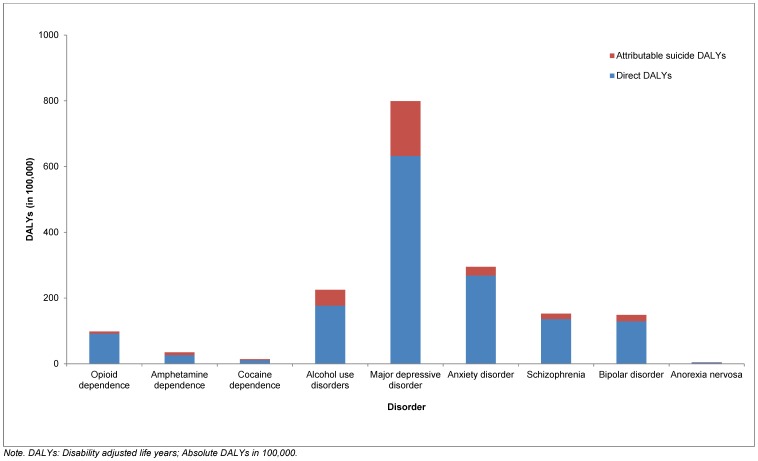
Suicide DALYs attributable to mental and substance use disorders by disorder, in 2010.

Of the suicide DALYs attributable to mental and substance use disorders, MDD was responsible for the largest proportion (46.1% (28.0%–60.8%)), followed by alcohol dependence (13.25% (12.0%–15.0%)), anxiety disorder (7.4% (3.0%–12.7%)), bipolar disorder (5.4% (1.8%–10.7%)), schizophrenia (4.7% (4.1%–5.3%)), amphetamine dependence (2.4% (0.9%–4.6%)), opioid dependence (1.9% (1.1%–2.9%)), cocaine dependence (0.9% (0.3%–1.8%)) and anorexia nervosa (0.2% (0.02%–0.5%)) (figure 4). MDD explained the most suicide DALYs and anorexia nervosa the least across all age groups, sex and regions although most of the age and regional differences between disorders remained within wide and overlapping confidence intervals (Table S6 in File S1).

The additional burden attributable to suicide for each mental and substance use disorder (over and above the DALYs assigned to them as a direct cause) is also illustrated in figure 4. The inclusion of attributable suicide burden increased the fatal burden (YLLs) due to mental and substance use disorders from 0.5% (0.4%–0.7%) (assigned to them as a direct cause) to 1.8% (1.4%–2.2%) of global YLLs and the overall burden (DALYs) of mental and substance use disorders from 7.4% (6.2%–8.6%) to 8.3% (7.1%–9.6%) of global DALYs. Out of the 10 leading classes of diseases included in GBD 2010 [5], mental and substance use disorders increased from the 5^th^ to the 3^rd^ leading class of disease burden once the burden attributable to suicide was considered; exceeding the burden due to neoplasms (7.6% (7.0%–8.2%) of global DALYs) and neonatal conditions (8.1% (7.3%–9.0%) of global DALYs) but not cardiovascular and circulatory diseases (11.9% (11.1%–12.7%) of global DALYs) and diarrhea, LRI, meningitis, and other common infectious diseases (11.4% (10.4%–12.8%) of global DALYs). The global DALY ranking of individual disorders (as presented in GBD 2010's publication series [5]) also increased when attributable suicide burden was included (table 2). Although within overlapping ranges of uncertainty, the ranking for alcohol dependence increased the most, from the 35^th^ (29^th^–45^th^) to the 28^th^ (26^th^–37^th^) leading cause of burden.

**Table 2:** Global DALY proportions and rankings before and after the addition of attributable suicide burden, in 2010.

	After addition of attributable suicide burden (95% UI)			
Disorder	Direct DALYs	Mean rank	Direct plus attributable DALYs	Mean rank
	As a proportion of total DALYs		As a proportion of total DALYs	
Major depressive disorder^a^	2.5% (1.9%–3.3%)	11 (7–14)	3.2% (2.5%–4.0%)	8 (4–11)
Anxiety disorder^a^	1.1% (0.8%–1.5%)	26 (19–33)	1.2% (0.9%–1.6%)	25 (17–30)
Alcohol dependence^a^	0.7% (0.5%–0.9%)	35 (29–45)	0.9% (0.7%–1.1%)	28 (26–37)
Schizophrenia^a^	0.6% (0.4%–0.7%)	43 (36–57)	0.7% (0.5%–0.9%)	39 (30.5–50)
Bipolar disorder^a^	0.5% (0.3%–0.8%)	46 (35–59)	0.6% (0.4%–0.8%)	44 (31–56)
Mental and substance use disorders combined^b^	7.4% (6.2%–8.6%)	5 (3–6)	8.3% (7.1%–9.6%)	3 (3–6)

*Note. DALYs: Disability adjusted life years; 95% UI: 95% uncertainty interval;*

a *Global ranking of direct burden for each disorder was from the official GBD 2010 disease ranking for 2010* [*5*] *. Illicit drug use disorders have not been included here as the GBD 2010 official disease ranking investigated drug use disorders as group (rather than by specific drug types). Similarly, the ranking for anorexia nervosa was presented in addition to bulimia nervosa;*

b *The global ranking of direct burden of mental and substance use disorders as a group compares the direct burden of the 11 main classes of diseases in GBD 2010* [*10*] *.*

## Discussion

Mental and substance use disorders are associated with an increased risk of suicide, a finding that is well established in the literature [19], [20], [27] but until now, not quantified in terms of a global comparison of disease burden. DALY rankings in GBD 2010 were based on a classification of mutually exclusive disease and injury categories [5], [10]. Considering the additional burden due to mental and substance use disorders as a risk factor for suicide elevated mental and substance use disorders from the fifth to the third leading disease category of global burden in 2010. Few mental and substance use disorders are recognized as a primary cause of death in mortality registrations, and those that are recognised are often under-represented. The data presented here provide a more comprehensive insight into the magnitude of the burden due to these disorders.

Mental and substance use disorders were the cause of two-thirds of all suicide DALYs reported in GBD 2010. Aside from emphasising these as a debilitating group of disorders, our findings highlight the importance of prioritising the prevention, early detection and effective management of mental and substance use disorders – particularly MDD – as a key suicide prevention strategy. Presenting the differences in attributable burden between regions and countries also provides a beginning for developing policies or intervention strategies that are applicable at the national level. Such interventions can be described as ‘selective’, in the sense that they target subgroups of the population whose members have yet to manifest suicidal behaviours, but exhibit risk factors (in this case, mental and substance use disorders) that predispose them to do so in the future. These can be contrasted with ‘universal’ interventions, which target whole populations with the aim of favorably shifting proximal and distal risk (and protective) factors across the entire population, and ‘indicated interventions’ which are designed for individuals already exhibiting suicidal behaviours [51].

Typically, countries that have put in place national suicide prevention strategies have funded a range of universal, selective and indicated interventions, in recognition of the variety of risk and protective factors associated with suicide [52]. However our findings suggest that a relatively greater emphasis on selective interventions targeting individuals with mental and substance use disorders may be applicable. By way of example, equipping general practitioners to detect, diagnose and manage MDD is likely to have benefits, particularly because many individuals with MDD will receive care from a general practitioner rather than a specialist mental health provider. This was one of the few interventions for which there was good evidence of effectiveness as a suicide prevention strategy in a recent review by Mann and colleagues [53]. That said, ensuring that care from general practitioners is evidence-based requires further consideration, given findings that rates of minimally adequate treatment for depression are lower among patients treated solely by general practitioners or in the general medical care sector, compared to those treated by specialist mental health providers [54], [55].

However universal and indicated interventions have their place, particularly in low and middle income countries where mental and substance use disorders were associated with a lesser proportion of the burden of suicide. In these countries, universal interventions for example restricting access to means (e.g., pesticides) is worth pursuing given that they are relatively cheap to implement, can have a broad community reach and are known to be effective [53].

Although within overlapping bounds of uncertainty, we found that attributable suicide DALY rates among young people aged 15–19 years were approaching those of the adult age groups. Although males had higher rates of attributable burden in most age groups, female rates were higher between the ages of 10 and 19 years. These age-related findings support the importance of school-based prevention programs which include a focus on mental health targeted to at-risk adolescents. The sex-difference in attributable burden also needs to be considered when formulating prevention strategies for this age group. Although evidence of a reduction in suicide behaviours has not been demonstrated, there is evidence for the effectiveness of school-based programs in reducing the effect of risk factors such as depression [52], [56]. A recent systematic review of interventions targeting adolescents or young adults at risk of suicide identified individual cognitive behavioral therapy-based interventions and attachment-based family therapy as promising interventions, requiring further investigation [57].

As there was insufficient data to (1) obtain pooled RR estimates for all countries or regions included in GBD 2010 and (2) clearly detect differences in RR estimates between all countries/regions, the pooled RR estimates used to estimate PAFs were assumed to be constant across age, sex and country. Instead, the variation in attributable DALYs across countries was driven by (a function of both) the prevalence of mental and substance use disorders and the amount of burden accounted for by suicide in each country. In addition, given evidence for differences in the underlying causes of suicide in China, India and Taiwan [45]–[48], where it has been well documented that the ease of availability of particularly lethal means of self-harm such as pesticides may influence patterns of suicide, we constrained the maximum proportion of suicide attributable to mental and substance use disorders to a ceiling value of 68.3%. In spite of this, some Asian countries were amongst those with the highest rates of attributable suicide burden due to the high rates of suicide in those countries. This emphasizes the fact that although there may be other risk factors for suicide, the prioritisation of mental and substance use disorders in the prevention of suicide remains a global priority.

The maximum proportion of suicide attributable to mental and substance use disorders in all other countries was constrained to a ceiling value of 84.5%. The studies categorized as “all other countries” were mainly from North America, Western Europe and Australia and, although we had data for three low to middle income countries (Colombia, Pakistan and Indonesia), this pooled proportion might not be appropriate for use in Sub-Saharan Africa where we found no data. It is possible that these countries have a different distribution of suicides attributable to mental and substance use disorders but more cross-national RR data are required before we can incorporate this in our findings. Islamic countries, for instance from North Africa/Middle East, were amongst the countries with the lowest proportion of attributable burden, despite being allocated the higher ceiling value of 84.5%. In contrast to the high rates of depression in the Middle East, rates of suicide were low. The lowest rate of suicide recorded in GBD 2010 was from Saudi Arabia. Stigma around suicide due to religious beliefs and legislative prohibition (i.e. suicide being considered as a criminal offence) can lead to fewer cases of suicide being recorded as a cause of death in countries from the Middle East. For similar reasons, the degree of psychopathology underpinning suicide cannot be as clearly assessed in these countries [58], [59]. These issues may have biased our estimates of attributable burden. The large bounds of uncertainty presented reflect this to some extent; however, more data are required on the distribution and aetiology of suicide in these countries to improve estimates.

Like all population-based analyses, a number of methodological limitations need to be considered here. The ceiling values for suicide attributable to mental and substance use disorders were derived from psychological autopsy studies. As these collect retrospective data after the individual had died, they are limited by the accuracy of coroners' reports and systematic bias from interviewees [49]. Although the pooled RR estimates used were derived from more representative population-based prospective cohort studies, there were only a few estimates available for most disorders. We applied the same pooled RR across all countries, sex and age groups for each disorder to reduce errors in estimates as a result of paucity in the data. It is possible that this masked differences in the distribution of attributable suicide DALYs. More representative population cohort studies are now emerging from low and middle income countries such as India [16]. We hope that the scrutiny of data presented here will encourage more and better quality data collection for mental and substance use disorders as risk factors for suicide. Until then, it is important to consider the uncertainty around our final estimates in interpreting these findings.

CRA methodology assumes a causal relationship between the exposure and outcome [7]. In support for this, the RR estimates used here showed that mental and substance use disorders were significantly associated with suicide risk, even when other risk factors such as socio-economic factors (e.g. adverse marital, employment and socio-economic status) were considered [20], [21]. Another assumption was that the proportion of suicide burden attributable to mental and substance use disorders was estimated while holding all other independent risk factors constant. We estimated the joint effect of all mental and substance use disorders on suicide while adjusting for comorbidity between these disorders, the next step would be to explore the joint effect of mental and substance use disorder with other risk factors of suicide. Finally, PAF calculations were sensitive to the exposure distribution used. Here we used DisMod-MR to pool the prevalence of each disorder based on the raw epidemiological data that were available [10], [38]. Although this provided consistent prevalence estimates by country, region, age, sex, and year, in some cases DisMod-MR was required to adjust for considerable heterogeneity in the raw data. This was, to some extent, incorporated in our analyses through the 95% uncertainty intervals around all prevalence estimates propagated to the final attributable burden estimates.

## Conclusions

Mental and substance use disorders were responsible for two thirds of the suicide burden in 2010, adding a further 22 million DALYs to their global burden. More consideration needs to be given to interventions targeted to populations with, or at risk for, mental and substance use disorders as an effective strategy for suicide prevention.

## Supporting Information

File S1 **This file contains Text S1 and Tables S1 to S6.**
(ZIP)
