# Supplementary material for: The Burden Attributable to Mental and Substance Use Disorders as Risk Factors for Suicide: Findings from the Global Burden of Disease Study 2010
Source: PLoS One. 2014 Apr 2;9(4):e91936. doi: 10.1371/journal.pone.0091936 (PMC3973668; doi:10.1371/journal.pone.0091936)
Supplement: File S1 — This file contains Text S1 and Tables S1 to S6. (ZIP) [file pone.0091936.s001.zip › Supplemental files/Table S1_Ferrari et al_181013.docx]

### Table S1: Summary of studies reporting the relative-risk of suicide in those with mental and substance use disorders.

| Data source | Country | Disorder | Age Range  (years) | Epoch Range | Sex^a^ | Relative-risk  (95% UI) | Quality score/1^c^ |
| --- | --- | --- | --- | --- | --- | --- | --- |
| Mental disorders |  |  |  |  |  |  |  |
| Shaffer et al., 1996 [[1](#_ENREF_1)] | USA | Major Depression  Bipolar disorder  Anxiety disorders | 0-19 | 1984-1986 | M F  M F  M F | 16.1 (2.0-128.1) -  - 1.6 (0.1-26.5)  2.6 (1.1-6.0) 0.7 (0.2-3.3) | 0.8 |
| Lesage et al., 1994 [[2](#_ENREF_2)] | Canada | Major depression  Major depression with psychotic features  Bipolar disorder  Bipolar NOS  Depression NOS  Generalized anxiety disorder  Panic Disorder  Agoraphobia  Obsessive compulsive disorder  Social phobia  Somatoform disorder  Anxiety NOS  Schizophrenia  Schizoaffective disorder  Schizophrenia NOS | 18-35 | 1987-1989 | M  M  M  M  M  M  M  M  M    M  M  M  M M  M  M | 11.2 (3.7-33.9)  -  1.0 (0.2-5.1)  1 (0.1-16.3)  -  3.1 (0.3-30.3)  -  -  2.0 (0.2-22.8)    1.0 (0.1-16.3)  2.0 (0.2-22.8)  3.1 (0.3-30.3)  1.4 (0.3-6.3) -  -  - | 0.6 |
| Waern et al., 2002 [[2](#_ENREF_2)] | Sweden | Major depression  Minor depression  Anxiety disorders | 65-99 | 1994-1996 | M F  M F  M F | 34.4 (7.5-157.1) 28.7 (6.2-134.1)  5.7 (1.4-22.6) -  2.6 (0.5-12.0) 6.6 (1.7-26.1) | 0.8 |
| Dutta et al., 2007 [[3](#_ENREF_3)] | United Kingdom | Bipolar I disorder | 16-99 | 1965-1999 | M  F | 12.76(5.13-26.29)  4.27(0.11-23.78) | 0.9 |
| Brent et al., 1999 [[4](#_ENREF_4)] | USA | Anxiety disorders | 13-19 | 1989-1994 | M F | 13.0 (1.7-100.2) 2.8 (0.7-11.9) | 0.8 |
| Kreipe et al., 1989 [[5](#_ENREF_5)] | USA | Anorexia Nervosa | 12-19 | 1979-1984 | F | 20.41 (0.5-113.7) | 0.6 |
| Keel et al., 2003 [[6](#_ENREF_6)] | USA | Anorexia Nervosa | 12-99 | 1987-2000 | F | 29.41 (8.0-75.3) | 0.8 |

| Data source | Country | Disorder | Age Range  (years) | Epoch Range | Sex^a^ | Relative-risk  (95% uncertainty interval) | Quality score/1^c^ |
| --- | --- | --- | --- | --- | --- | --- | --- |
| Mental disorders |  |  |  |  |  |  |  |
| Korndofer et al., 2003 [[7](#_ENREF_7)] | USA | Anorexia Nervosa | 10-57 | 1935-1989 | M F | - 10.36 (1.3-37.4) | 0.9 |
| Zipfel et al., 2000 [[8](#_ENREF_8)] | Germany | Anorexia Nervosa | 0-99 | 1974-1998 | F | 23.81 (2.9-86.0) | 0.8 |
| Papadopoulos et al., 2009 [[9](#_ENREF_9)] | Sweden | Anorexia Nervosa | 10-40 | 1973-2003 | F | 13.98 (11.2-17.3) | 0.8 |
| Moller-Madsen, 1998 [[10](#_ENREF_10)] | Denmark | Anorexia Nervosa | 0-99 | 1970-1994 | M F | 31.75 (3.8-114.7) 20.25 (11.6-32.9) | 0.9 |
| Signorini et al., 2007 [[11](#_ENREF_11)] | Italy | Anorexia Nervosa | 10-52 | 1994-2003 | F | 6.8 (0.2-37.9) | 0.8 |
| Qin & Nordentoft, 2005 [[12](#_ENREF_12)] | Denmark | Schizophrenia | 0-99 | 1981-1997 | M F | 11.8 (10.9-12.8) 12.6 (11.4-13.9) | 0.8 |
| Riala et al., 2007 [[13](#_ENREF_13)] | Finland | Schizophrenia | 0-33 | ͌͌1966-2001 | M | 13.7 (5.2-36.1) 19.5 (4.2-90.8) | 0.9 |
| Illicit drug use disorders | |  |  |  |  |  |  |
| Pavarin, 2008 [[14](#_ENREF_14)] | Italy | Cocaine dependence | 0-99 | 1989-2004 | P | 10.3(00.01-32.2) | - |
| Tyndall et al., 2001 [[15](#_ENREF_15)] | Canada | Cocaine dependence | 14-61 | 1996-2004 | P | 15.1(4.01-31.0) | - |
| Fugelstad et al., 1997 [[16](#_ENREF_16)] | Sweden | Amphetamine dependence  Opioid dependence | 0-99 | 1985-1992 | P | 4.3(1.1-8.6)  13.9(10.6-30.3) | - |
| Stenbacka et al., 2007 [[17](#_ENREF_17)] | Sweden | Opioid dependence | 14-47 | 1967-2003 | P | 8.03(5.6-10.7) | - |
| Miller et al., 2007 [[18](#_ENREF_18)] | Canada | Opioid dependence | 0-29 | 1996-2004 | P | 10.1(0.01-25.1) | - |
| Wang et al., 2005 [[19](#_ENREF_19)] | USA | Cocaine dependence | 0-99 | 1988-2001 | P | 3.04(0.01-7.8) | - |
| Goldstein et al., 1995 [[20](#_ENREF_20)] | USA | Opioid dependence | 13-60 | 1969-1993 | P | 3.2(1.3-5.1) | - |
| Soyka et al., 2006 [[21](#_ENREF_21)] | Germany | Opioid dependence | 17-62 | 1993-1994 | P | 7.2(0.01-17.8) | - |
| Fugelstad et al., 1998 [[22](#_ENREF_22)] | Sweden | Opioid dependence | 20-99 | 1986-1993 | P | 13.9(0.01-38.5) | - |
| Antolini et al., 2006 [[23](#_ENREF_23)] | Italy | Opioid dependence | 0-99 | 1975-1999 | P | 6.7(4.3-9.5) | - |
| Brancato et al., 1995 [[24](#_ENREF_24)] | Italy | Opioid dependence | 18-38 | 1985-1994 | P | 18.3(0.01-58.04) | - |
| Galli & Musicco., 1994 [[25](#_ENREF_25)] | Italy | Opioid dependence | 14-57 | 1980-1991 | P | 6.7(2.6-11.6) | - |
| Manfredi et al., 2006 [[26](#_ENREF_26)] | Italy | Opioid dependence | 10-62 | 1977-2002 | P | 6.5(2.4-11.6) | - |
| Eskild et al., 1993 [[27](#_ENREF_27)] | Norway | Opioid dependence | 15-44 | 1985-1991 | P | 13.3(5.6-23.6) | - |
| Odegard et al., 2007 [[28](#_ENREF_28)] | Norway | Opioid dependence | 18-54 | 1981-2003 | P | 11.9(6.9-18.5) | - |
| Rossow., 1994 [[29](#_ENREF_29)] | Norway | Opioid dependence | 16-67 | 1961-1992 | P | 16.2(12.3-20.4) |  |
| Risser et al., 2001 [[30](#_ENREF_30)] | Austria | Opioid dependence | 0-99 | 1995-1997 | P | 5.7(0.01-16.8) | - |
| Bartu et al., 2004 [[31](#_ENREF_31)] | Australia | Opioid dependence | 18-50 | 1985-1998 | P | 1.5(1.1-1.97) | - |

| Data source | Country | Disorder | Age Range  (years) | Epoch Range | Sex^a^ | Relative-risk  (95% uncertainty interval) | Quality score/1^c^ |
| --- | --- | --- | --- | --- | --- | --- | --- |
| Illicit drug use disorders | |  |  |  |  |  |  |
| Degenhardt et al., 2009 [[32](#_ENREF_32)] | Australia | Opioid dependence | 20-40 | 1985-2005 | P | 4.98(4.5-5.5) | - |
| Digiusto et al., 2004 [[33](#_ENREF_33)] | Australia | Opioid dependence | 0-99 | 1998-2202 | P | 13.6(0.01-47.3) | - |
| Tait et al., 2008 [[34](#_ENREF_34)] | Australia | Opioid dependence | 18-35 | 1997-2002 | P | 3.9(1.3-7.3) | - |
| Vlahov et al., 2005 [[35](#_ENREF_35)] | USA | Opioid dependence | 0-99 | 1988-2005 | P | 2.7(0.01-8.2) | - |
| Vlahov et al., 2008 [[36](#_ENREF_36)] | USA | Opioid dependence |  |  |  | 3.9(1.3-7.3) | - |
| Oppenheimer et al., 1994 [[37](#_ENREF_37)] | United Kingdom | Opioid dependence | 17-52 | 1969-1981 | P | 4.8(0.01-12.6) | - |
| Alcohol dependence^c^ |  |  |  |  |  |  |  |
| Wilcox et al 2004^c^ [[38](#_ENREF_38)] | USA, Kuwait,  Sweden  Spain,  United Kingdom | Alcohol use disorders | - | - | P | 9.8 (8.98–10.7) |  |

*Note. NOS: Not otherwise specified; ^a^Sex: Males (M), Female (F), Persons(P). ^b^Quality scores calculated for mental disorders only* due to insufficient data for substance use*. Studies scored out of 8 where studies reporting gender specific estimates =2 and person estimates only=1; studies derived from population representative samples= 2 and hospitalised samples=1; studies covering the entire lifespan=2 and only a specific age group=1; studies using a prospective design=2 and a retrospective design=1. RR estimate for alcohol dependence obtained from an existing literature review and meta-analysis of 12 studies [39]. Due to paucity of data estimates based on clinical samples were also included for bipolar disorder and anorexia nervosa. The difference in representativeness of each sample was reflected in the quality indices.*

**References**

1. Shaffer D, Gould MS, Fisher P, Trautman P, Moreau D, et al. (1996) Psychiatric diagnosis in child and adolescent suicide. Archives of General Psychiatry 53: 339-348.

2. Lesage AD, Boyer R, Grunberg F, Vanier C, Morisette R, et al. (1994) Suicide and mental disorders: a case–control study of young men. American Journal of Psychiatry 151: 1063–1068.

3. Dutta R, Boydell J, Kennedy N, J VANO, Fearon P, et al. (2007) Suicide and other causes of mortality in bipolar disorder: a longitudinal study. Psychol Med 37: 839-847.

4. Brent DA, Baugher M, Bridge J, Chen T, Chiappetta L (1999) Age and sex related risk factors for adolescent suicide

Journal of the American Academy of Child Adolescent Psychiatry 38 1497-1505.

5. Kreipe RE, Churchill BH, Strauss J (1989) Long-term outcome of adolescents with anorexia nervosa. Am J Dis Child 143: 1322-1327.

6. Keel PK, Dorer DJ, Eddy KT, Franko D, Charatan DL, et al. (2003) Predictors of mortality in eating disorders. Arch Gen Psychiatry 60: 179-183.

7. Korndorfer SR, Lucas AR, Suman VJ, Crowson CS, Krahn LE, et al. (2003) Long-term survival of patients with anorexia nervosa: a population-based study in Rochester, Minn. Mayo Clin Proc 78: 278-284.

8. Zipfel S, Lowe B, Reas DL, Deter HC, Herzog W (2000) Long-term prognosis in anorexia nervosa: lessons from a 21-year follow-up study. Lancet 355: 721-722.

9. Papadopoulos FC, Ekbom A, Brandt L, Ekselius L (2009) Excess mortality, causes of death and prognostic factors in anorexia nervosa. Br J Psychiatry 194: 10-17.

10. Moller-Madsen SM, Nystrup J, Nielsen S (1998) [Mortality of anorexia nervosa in Denmark 1970-1987]. Ugeskr Laeger 160: 5509-5513.

11. Signorini A, De Filippo E, Panico S, De Caprio C, Pasanisi F, et al. (2007) Long-term mortality in anorexia nervosa: a report after an 8-year follow-up and a review of the most recent literature. European Journal of Clinical Nutrition 61: 119-122.

12. Qin P, Nordentoft M (2005) Suicide risk in relation to psychiatric hospitalization: evidence based on longitudinal registers. Arch Gen Psychiatry 62: 427-432.

13. Riala K, Alaräisänen A, Taanila A, Hakko H, Timonen M, et al. (2007) Regular daily smoking among 14-year-old adolescents increases the subsequent risk for suicide: the Northern Finland 1966 Birth Cohort Study. The Journal of clinical psychiatry 68: 775-780.

14. Pavarin RM (2008) Cocaine consumption and death risk: a follow-up study on 347 cocaine addicts in the metropolitan area of Bologna. Ann Ist Super Sanita 44: 91-98.

15. Tyndall MW, Craib KJ, Currie S, Li K, O'Shaughnessy MV, et al. (2001) Impact of HIV infection on mortality in a cohort of injection drug users. J Acquir Immune Defic Syndr 28: 351-357.

16. Fugelstad A, Annell A, Rajs J, Agren G (1997) Mortality and causes and manner of death among drug addicts in Stockholm during the period 1981-1992. Acta Psychiatr Scand 96: 169-175.

17. Stenbacka M, Leifman A, Romelsjo A (2007) Mortality among opiate abusers in Stockholm: a longitudinal study. . Heroin Addiction and Related Clinical Problems 9: 41-49.

18. Miller CL, Kerr T, Strathdee SA, Li K, Wood E (2007) Factors associated with premature mortality among young injection drug users in Vancouver. Harm Reduct J 4: 1.

19. Wang C, Vlahov D, Galai N, Cole SR, Bareta J, et al. (2005) The effect of HIV infection on overdose mortality. AIDS 19: 935-942.

20. Goldstein A, Herrera J (1995) Heroin addicts and methadone treatment in Albuquerque: a 22-year follow-up. Drug Alcohol Depend 40: 139-150.

21. Soyka M, Apelt SM, Lieb M, Wittchen HU (2006) One-year mortality rates of patients receiving methadone and buprenorphine maintenance therapy: a nationally representative cohort study in 2694 patients. J Clin Psychopharmacol 26: 657-660.

22. Fugelstad A, Agren G, Romelsjo A (1998) Changes in mortality, arrests, and hospitalizations in nonvoluntarily treated heroin addicts in relation to methadone treatment. Subst Use Misuse 33: 2803-2817.

23. Antolini G, Pirani M, Morandi G, Sorio C (2006) [Gender difference and mortality in a cohort of heroin users in the Provinces of Modena and Ferrara, 1975-1999]. Epidemiol Prev 30: 91-99.

24. Brancato V, Delvecchio G, Simone P (1995) [Survival and mortality in a cohort of heroin addicts in 1985-1994]. Minerva Med 86: 97-99.

25. Galli M, Musicco M (1994) Mortality of intravenous drug users living in Milan, Italy: role of HIV-1 infection. COMCAT Study Group. AIDS 8: 1457-1463.

26. Manfredi R, Sabbatani S, Agostini D (2006) Trend of mortality observed in a cohort of drug addicts of the metropolitan area of Bologna, North-Eastern Italy, during a 25-year-period. Coll Antropol 30: 479-488.

27. Eskild A, Magnus P, Samuelsen SO, Sohlberg C, Kittelsen P (1993) Differences in mortality rates and causes of death between HIV positive and HIV negative intravenous drug users. Int J Epidemiol 22: 315-320.

28. Odegard E, Amundsen EJ, Kielland KB (2007) Fatal overdoses and deaths by other causes in a cohort of Norwegian drug abusers--a competing risk approach. Drug Alcohol Depend 89: 176-182.

29. Rossow I (1994) Suicide among drug addicts in Norway. Addiction 89: 1667-1673.

30. Risser D, Honigschnabl S, Stichenwirth M, Pfudl S, Sebald D, et al. (2001) Mortality of opiate users in Vienna, Austria. Drug Alcohol Depend 64: 251-256.

31. Bartu A, Freeman NC, Gawthorne GS, Codde JP, Holman CD (2004) Mortality in a cohort of opiate and amphetamine users in Perth, Western Australia. Addiction 99: 53-60.

32. Degenhardt L, Randall D, Hall W, Law M, Butler T, et al. (2009) Mortality among clients of a state-wide opioid pharmacotherapy program over 20 years: risk factors and lives saved. Drug Alcohol Depend 105: 9-15.

33. Digiusto E, Shakeshaft A, Ritter A, O'Brien S, Mattick RP, et al. (2004) Serious adverse events in the Australian National Evaluation of Pharmacotherapies for Opioid Dependence (NEPOD). Addiction 99: 450-460.

34. Tait RJ, Ngo HT, Hulse GK (2008) Mortality in heroin users 3 years after naltrexone implant or methadone maintenance treatment. J Subst Abuse Treat 35: 116-124.

35. Vlahov D, Galai N, Safaeian M, Galea S, Kirk GD, et al. (2005) Effectiveness of highly active antiretroviral therapy among injection drug users with late-stage human immunodeficiency virus infection. Am J Epidemiol 161: 999-1012.

36. Vlahov D, Wang C, Ompad D, Fuller CM, Caceres W, et al. (2008) Mortality risk among recent-onset injection drug users in five U.S. cities. Subst Use Misuse 43: 413-428.

37. Oppenheimer E, Tobutt C, Taylor C, Andrew T (1994) Death and survival in a cohort of heroin addicts from London clinics: a 22-year follow-up study. Addiction 89: 1299-1308.

38. Wilcox HC, Conner KR, Caine ED (2004) Association of alcohol and drug use disorders and completed suicide: an empirical review of cohort studies. Drug Alcohol Depend 76 Suppl: S11-19.
