# Supplementary material for: The Burden Attributable to Mental and Substance Use Disorders as Risk Factors for Suicide: Findings from the Global Burden of Disease Study 2010
Source: PLoS One. 2014 Apr 2;9(4):e91936. doi: 10.1371/journal.pone.0091936 (PMC3973668; doi:10.1371/journal.pone.0091936)
Supplement: File S1 — This file contains Text S1 and Tables S1 to S6. (ZIP) [file pone.0091936.s001.zip › Supplemental files/Table S2_Ferrari et al_181013.docx]

### Table S2: Pooled relative-risk associated to mental and substance use disorders as a risk factor for suicide.

|  | **Pooled Relative-risk (95% UI)** | | |
| --- | --- | --- | --- |
| **Disorder** | **Overall** | **Male** | **Female** |
| Major Depressive disorder | QE: 19.9 (9.5-41.7)  RE:18.6 (9.02-38.5) | QE: 17.7 (7.6-41.2)  RE:16.5 (7.2-37.5) | QE: 28.7 (6.2-133.5)  RE:28.7 (6.2-133.5) |
| Bipolar Disorder | QE: 5.7 (2.6-12.4)  RE: 2.9 (0.7-11.7) | QE:6.3 (2.6-15.2)  RE: 2.8 (0.4-20.9) | QE: 2.8 (0.1-19.2)  RE:2.7 (0.4-18.4) |
| Anxiety Disorder | QE: 2.7 (1.7-4.3)  RE:2.7 (1.7-4.3) | QE:2.8 (1.6-5.2)  RE:2.8 (1.6-5.02) | QE: 2.4 (1.0-5.6)  RE:2.4 (0.7-8.6) |
| Anorexia Nervosa | QE: 7.57 (2.24-25.62)  RE:6.9 (4.1-11.5) | QE:6.17 (3.00-12.65)  RE:6.2 (2.8-11.8) | QE: 8.63 (1.69-43.93)  RE:7.7 (3.7-15.9) |
| Schizophrenia | QE: 12.6 (11.01-14.5)  RE:12.1 (11.4-12.9) | QE: 12.04 (10.3-14.03)  RE:11.8 (10.9-12.8) | QE: 13.4 (10.6-16.8)  RE:12.6 (11.4-13.9) |
| Alcohol dependence^a^ | RE: 9.8 (8.98–10.7) | RE: 4.8 (4.4–5.2) | RE: 16.9 (12.5–22.4) |
| Cocaine dependence | RE: 16.9 (6.01-47.2) | - | - |
| Opioid dependence | RE: 6.9 (4.5-10.5) | - | - |
| Amphetamine dependence | RE: 4.5 (1.1-9.03) | - | - |

*Note**. 95% UI: 95% uncertainty interval; QE: Quality effects model estimate; RE: Random effects model estimate; ^a^Estimates for alcohol dependence were extracted from Wilcox et al* *[*[*1*](#_ENREF_1)*]; There was sufficient data to calculate quality effects estimates for mental disorders only. There insufficient data to calculate sex specific estimates for illicit drug use disorders.*

**References**

1. Wilcox HC, Conner KR, Caine ED (2004) Association of alcohol and drug use disorders and completed suicide: an empirical review of cohort studies. Drug Alcohol Depend 76 Suppl: S11-19.
