# Supplementary material for: The Burden Attributable to Mental and Substance Use Disorders as Risk Factors for Suicide: Findings from the Global Burden of Disease Study 2010
Source: PLoS One. 2014 Apr 2;9(4):e91936. doi: 10.1371/journal.pone.0091936 (PMC3973668; doi:10.1371/journal.pone.0091936)
Supplement: File S1 — This file contains Text S1 and Tables S1 to S6. (ZIP) [file pone.0091936.s001.zip › Supplemental files/Table S3_Ferrari et al_181013.docx]

### Table S3: Summary of studies reporting the proportion of suicide cases attributable to mental and substance use disorders.

| **Data source** | **Country** | **Epoch range** | **Age range** | **Sex^a^** | **Proportion %^b^** | **Quality Score^c^ /1** | **Included Disorders** |
| --- | --- | --- | --- | --- | --- | --- | --- |
| Foster et al., 1997 [[1](#_ENREF_1)] | Ireland | 1992-1993 | 14-99 | M  F | 82.8  100 | 0.9 | Mood, Substance, Psychotic, Anxiety, Somatoform, Organic Adjustment, Other axis l disorders |
| Schneider et al., 2005 [[2](#_ENREF_2)] | Germany | 1999-2000 | 0-99 | M  F | 90.4  88.1 | 0.7 | Mood, Substance, Psychotic disorders |
| Groholt et al., 1997 [[3](#_ENREF_3)] | Norway | 1990-1992 | 8-19 | M  F | 74.7  73.3 | 0.8 | Mood, Disruptive, Psychotic, Adjustment, Substance disorders |
| Henriksson et al., 1993 [[4](#_ENREF_4)] | Finland | 1987-1988 | 10-89 | M  F | 97.5  100 | 1.0 | Mood, Substance, Psychotic, Organic , Anxiety, Adjustment, Personality disorders |
| Asgard, 1990 [[5](#_ENREF_5)] | Denmark | 1982-1982 | 15-99 | F | 99.0 | 0.6 | Mood, Psychotic , Substance, Anxiety, Adjustment, Organic disorders |
| Runeson, 1989 [[6](#_ENREF_6)] | Sweden | 1984-1989 | 15-29 | P | 98.3 | 0.4 | Mood, Eating, Dementia, Substance, Psychotic , Anxiety, Somatization, Adjustment, Personality disorders |
| Waern et al., 2002 [[7](#_ENREF_7)] | Sweden | 1994-1996 | 65-97 | P | 96.5 | 0.4 | Mood, Substance, Anxiety, Psychotic disorders, dementia |
| Boardman et al., 1999 [[8](#_ENREF_8)] | UK | 1991-1995 | 14-89 | P | 71.2 | 0.6 | Mood, Psychotic, Substance, Organic , Personality disorders, |
| Cavanagh et al., 1999 [[9](#_ENREF_9)] | UK | 1996-1998 | N/S | P | 97.8 | 0.6 | Mood, Psychotic, Anxiety, Substance, Personality disorders |
| Harwood et al., 2001 [[10](#_ENREF_10)] | UK | 1995-1998 | 60-99 | P | 77.0 | 0.6 | Mood, Psychotic, Substance-related, Schizophrenia, Sexual, Adjustment, Somatoform, Organic, Personality disorders |
| Houston et al., 2001 [[11](#_ENREF_11)] | UK | 1993-1995 | 15-24 | P | 70.4 | 0.4 | Mood, Psychotic, Substance, Anxiety, Somatoform, Gender identity, Eating disorders |
| Appleby et al., 1999 [[12](#_ENREF_12)] | UK | 1995-1996 | 13-35 | P | 90.5 | 0.3 | N/S |

| **Data source** | **Country** | **Epoch range** | **Age range** | **Sex^a^** | **Proportion %^b^** | **Quality Score^c^ /1** | **Included Disorders** |
| --- | --- | --- | --- | --- | --- | --- | --- |
| Portzky et al., 2009 [[13](#_ENREF_13)] | Belgium | 1997-2001 | 15-19 | P | 100 | 0.4 | Mood, Psychotic, Anxiety, Substance, Adjustment Hyperkinetic, Aspergers, Eating, Impulsive, Gender identity, Conduct, Body dysmorphic disorders |
| Pompili et al., 2008 [[14](#_ENREF_14)] | Italy | 1994-2004 | 15-96 | P | 48.9 | 0.3 | Mood, Substance, other disorders |
| Isometsa et al., 1997 [[15](#_ENREF_15)] | Finland | 1987-1988 | 10-89 | P | 99.1 | 0.8 | Mood, Substance, Psychotic, Organic , Anxiety, Adjustment, Personality, other axis I and II disorders |
| Almasi et al., 2009 [[16](#_ENREF_16)] | Hungary | 2002-2004 | 30-62 | P | 69.1 | 0.6 | Mood, Psychotic, Anxiety, Eating disorders |
| Arato et al., 1988 [[17](#_ENREF_17)] | Hungary | 1985-1985 | 0-99 | M  F | 75.7  61.9 | 0.7 | Mood, Somatisation, Psychotic, Substance disorders |
| Zonda, 2006 [[18](#_ENREF_18)] | Hungary | N/S-N/S | N/S | P | 81.0 | 0.2 | Mood, Substance disorders |
| Brent et al., 1999 [[19](#_ENREF_19)] | USA | 1989-1991 | 13-19 | M  F | 82.4  81.0 | 0.7 | Mood, Anxiety, Substance, Conduct/ Antisocial disorders |
| Shaffer et al., 1996 [[20](#_ENREF_20)] | USA | 1984-1986 | 0-20 | M  F | 90.4  92.0 | 0.8 | Mood, Psychotic, Anxiety, Substance, Adjustment, Disruptive , Eating disorders |
| Conwell et al., 1991 [[21](#_ENREF_21)] | USA | 1987-1988 | 50-92 | M  F | 86.7  100 | 0.7 | Mood, Substance, Anxiety, Dementia/ Delirium disorders |
| Fowler et al., 1986 [[22](#_ENREF_22)] | USA | 1981-1983 | 10-29 | P | 86.5 | 0.3 | Mood, Substance, Conduct, Psychotic, Adjustment, Personality disorders |
| Rich et al., 1986 [[23](#_ENREF_23)] | USA | 1981-1983 | 0-99 | P | 93.5 | 0.6 | Mood, Psychotic, Organic, Substance, Adjustment, Child-adolescent, Axis II disorders |
| Shafii et al., 1988 [[24](#_ENREF_24)] | USA | NS-NS | 11-19 | P | 95.2 | 0.2 | Mood, Other disorders |
| Preville et al., 2005 [[25](#_ENREF_25)] | Canada | 1998-1999 | 60-99 | P | 42.1 | 0.4 | Mood, Anxiety, Substance disorders |
| Lesage et al., 1994 [[26](#_ENREF_26)] | Canada | 1987-1989 | 18-35 | M | 88.0 | 0.7 | Mood, Psychotic, Substance, Organic , Anxiety, Sexual, Somatoform, Childhood developmental, Disruptive, Personality disorders |
| McGirr et al., 2006 [[27](#_ENREF_27)] | Canada | 2000-2005 | 28-57 | M  F | 93.4  90.5 | 0.7 | Mood, Anxiety, Psychotic, Substance disorders |
| Palacioa et al., 2007 [[28](#_ENREF_28)] | Colombia | N/S-N/S | 19-42 | P | 89.8 | 0.3 | Mood, Substance, Psychotic, Adaptive, Personality disorders |
| Thacore et al., 2000 [[29](#_ENREF_29)] | Australia | 1992-1996 | 16-86 | P | 60.1 | 0.4 | Mood, Psychotic, Substance, Other disorders |

| **Data source** | **Country** | **Epoch range** | **Age range** | **Sex^a^** | **Proportion %^b^** | **Quality Score^c^ /1** | **Included Disorders** |
| --- | --- | --- | --- | --- | --- | --- | --- |
| Graham et al., 1992 [[30](#_ENREF_30)] | Australia | 1986-1988 | 15-59 | P | 57.1 | 0.6 | Mood, Psychotic, Organic, Substance, Conduct, Personality, Other disorders |
| Kurihara et al., 2009 [[31](#_ENREF_31)] | Indonesia | 2007-2007 | 13-87 | P | 80.0 | 0.6 | Mood, Psychotic, Anxiety, Substance, Adjustment disorders |
| Chen et al 2006 [[32](#_ENREF_32)] | China-Hong Kong | 2002-2004 | 15-59 | P | 80.7 | 0.4 | Mood, Substance, Other disorders |
| Chiu et al., 1994 [[33](#_ENREF_33)] | China-Hong Kong | 2000-2001 | 60-99 | M  F | 87.5  84.2 | 0.8 | Mood, Anxiety, Psychotic, Adjustment, Dementia, Somatoform, Substance disorders |
| Zhang et al., 2010 [[34](#_ENREF_34)] | China | 2005-2008 | 15-34 | M  F | 55.1  39.3 | 0.9 | Mood, Psychotic, Anxiety, Substance, Acute stress, Pathological gambling disorders |
| Zhang et al., 2009 [[35](#_ENREF_35)] | China | 2001-2003 | 0-99 | M  F | 72.9  55.6 | 0.8 | Mood, Psychotic, Anxiety, Substance disorders |
| Phillips et a., 2002 [[36](#_ENREF_36)] | China | 1995-2000 | 10-99 | P | 62.6 | 0.7 | N/S |
| Li et al., 2008 [[37](#_ENREF_37)] | China | 1995-2000 | 15-24 | P | 44.7 | 0.6 | N/S |
| Zhang et al, 2004 [[38](#_ENREF_38)] | China | 2001-2002 | N/S | P | 75.8 | 0.4 | Mood, Anxiety, Psychotic, Substance, Eating disorders |
| Cheng, 1995 [[39](#_ENREF_39)] | Taiwan | 1989-1991 | 15-99 | P | 98.3 | 0.9 | Mood, Organic, Psychotic, Substance, Mental retardation, Adjustment, Pathological gambling disorders |
| Vijayakumar et al., 1999 [[40](#_ENREF_40)] | India | 1994-1995 | 14-99 | P | 88.0 | 0.6 | Mood, Substance, Anxiety, Somatoform, Adjustment, Other disorders |
| Gururaj et al., 2004 [[41](#_ENREF_41)] | India | 2001-2002 | 0-99 | P | 42.8 | 0.4 | N/S |
| Khan et al., 2005 [[42](#_ENREF_42)] | India | 2003-2003 | 15-35 | P | 36.0 | 0.3 | N/S |
| Khan et al., 2008 [[43](#_ENREF_43)] | Pakistan | 2003-2003 | N/S | P | 96.0 | 0.8 | Mood, Psychotic , Adjustment, Acute stress reaction, Substance, Mental retardation, Personality disorders |

*Note. ^a^Sex: Males (M), Female (F), Persons(P); ^b^Proportion of suicide cases occurring as a result of a mental and substance use disorders; ^c^ Studies scored out of 9 where studies reporting male and female estimates =2, male or female estimates = 1, person estimates only=0; studies with a clearly reported sample and observation period = 2 and unreported sample and observation period =1; studies using national representative data =3, regionally-representative data =2, community-representative data=1; studies covering the entire lifespan=2 and only a specific age group=1.*

**References**

1. Foster T, Gillespie K, McClelland R (1997) Mental disorders and suicide in Northern Ireland. British Journal of Psychiatry 170 447-452.

2. Schneider B, Schnabel A, Webner B, Frolich L, Maurer K, et al. (2005) Nicotine use in suicide: a case-control study European Psychiatry 20: 129–136.

3. Groholt B, Ekeberg O, Wichstrom L, Haldorsen T (1997) Youth suicide in Norway, 1990–1992: a comparison between children and adolescents completing suicide and age- and gendermatched

controls. Suicide and Life Threatening Behavior 27: 250-263.

4. Henriksson MM, Aro HM, Marttunen MJ, Heikkinen ME, Isometsa ET, et al. (1993) Mental disorders and comorbidity in suicide American Journal of Psychiatry 150: 935–940.

5. Asgard U (1990) A psychiatric study of suicide among urban Swedish women Acta Psychiatrica Scandinavica 82: 115-124.

6. Runeson B (1989) Mental disorder in youth suicide. DSM-III-R Axes I and II. Acta Psychiatrica Scandinavica 79: 490-497.

7. Waern M, Runeson BS, Allebeck P, Beskow J, Rubenowitz E, et al. (2002) Mental disorder in elderly suicides: a case-control study American Journal of Psychiatry 159: 450-455.

8. Boardman AP, Grimbaldeston AH, Handley C, Jones PW, Willmott S (1999) The North Staffordshire Suicide Study: A case-control study of suicide in one health district Psychological Medicine 29: 27-33.

9. Cavanagh JT, Owens DG, Johnstone EC (1999) Life events in suicide and undetermined death in south-east Scotland: a case-control study using the method of psychological autopsy. Social Psychiatry and Psychiatric Epidemiology 34: 645-650.

10. Harwood D, Hawton K, Hope T, Jacoby R (2001) Psychiatric disorder and personality factors associated with suicide in older people: a descriptive and case-control study International Journal of Geriatric Psychiatry 16: 155-165.

11. Houston K, Hawton K, Shepperd R (2001) Suicide in young people aged 15–24: a psychological autopsy study Journal of Affective Disorders 63: 59-170.

12. Appleby L, Cooper J, Amos T, Faragher B (1999) Psychological autopsy study of suicides by people aged under 35 British Journal of Psychiatry 175: 168-174.

13. Portzky G, Audenaert K, Heeringen V (2009) Psychosocial and psychiatric factors associated with adolescent suicide: A caseecontrol psychological autopsy study. Journal of Adolescence 32: 849-862.

14. Pompili M, Innamorati M, Masotti V, Personne F, Lester D, et al. (2008) Suicide in the elderly: A psychological autopsy study in a North Italy Area (1994–2004). American Journal of Geriatric Psychiatry 16: 727-735.

15. Isometsa ET, Heikkinen ME, Henriksson MM, Marttunen MJ, Aro HM, et al. (1997) Differences between urban and rural suicides Acta Psychiatrica Scandinavica 95: 297–305.

16. Almasi K, Belso N, Kapur N, Webb R, Cooper J, et al. (2009) Risk factors for suicide in Hungary: a case-control study. BMC Psychiatry 9: 1-9.

17. Arato M, Demeter E, Rihmer Z, Somogyi E (1988) Retrospective psychiatric assessment of 200 suicides in Budapest. . Acta Psychiatrica Scandinavica 77 454-456.

18. Zonda T (2006) One-hundred cases of suicide in Budapest. A case–controlled psychological autopsy study. Crisis 27: 125–129.

19. Brent DA, Baugher M, Bridge J, Chen T, Chiappetta L (1999) Age and sex related risk factors for adolescent suicide

Journal of the American Academy of Child Adolescent Psychiatry 38 1497-1505.

20. Shaffer D, Gould MS, Fisher P, Trautman P, Moreau D, et al. (1996) Psychiatric diagnosis in child and adolescent suicide. Archives of General Psychiatry 53: 339-348.

21. Conwell Y, Olsen K, Caine ED, Flannery C (1991) Suicide in later life. Psychological autopsy findings International Psychogeriatrics

3: 59–66.

22. Fowler RC, Rich CL, Young D (1986) San Diego Suicide Study II. Substance abuse in young cases. Archives of General Psychiatry 43: 962–965.

23. Rich CL, Young D, Fowler RC (1986) San Diego suicide study. I. Young vs old subjects

Archives of General Psychiatry 43: 577-582.

24. Shafii M, Steltz-Lenarsky J, Derrick AM, Beckner C, Whittinghill JR (1988) Comorbidity of mental disorders in the post-mortem diagnosis of completed suicide in children and adolescents Journal of Affective Disorders

15 227-233.

25. Preville M, Hebert R, Boyer R, Bravo G, Seguin M (2005) Physical health and mental disorder in elderly suicide: a case-control study. Aging and mental health 9: 576–584.

26. Lesage AD, Boyer R, Grunberg F, Vanier C, Morisette R, et al. (1994) Suicide and mental disorders: a case–control study of young men. American Journal of Psychiatry 151: 1063–1068.

27. McGirr A, Séguin M, Renaud J, Benkelfat C, Alda M, et al. (2006) Gender and risk factors for suicide: evidence for heterogeneity in predisposing mechanisms in a psychological autopsy study. Journal of Clinical Psychiatry 67: 1012-1617.

28. Palacioa C, García J, Diago J, Zapata C, Lopez G, et al. (2007) Identification of suicide risk factors in Medellín, Colombia: A case-control study of psychological autopsy in a developing country. Archives of Suicide Research 11: 297-308.

29. Thacore VR, Varma SL (2000) A study of suicides in Ballarat, Victoria, Australia Crisis 21: 26-30.

30. Graham C, Burvill PW (1992) A study of coroner's records of suicide in young people, 1986–88 in Western Australia Australian and New Zealand Journal of Psychiatry 26: 30-39.

31. Kurihara T, Kato M, Reverger R, Tirta IG (2009) Risk factors for suicide in Bali: a psychological autopsy study. BMC Public Health 9: 1-7.

32. Chen EY, Chan WS, Wong PW, Chan SS, Chan CL, et al. (2006) Suicide in Hong Kong: a case-control psychological autopsy study. Psychol Med 36: 815-825.

33. Chiu HF, Yip PS, Chi I, Chan S, Tsoh J, et al. (2004) Elderly suicide in Hong Kong – a case controlled psychological autopsy study. Acta Psychiatrica Scandinavica 109: 299-305.

34. Zhang J, Xiao S, Zhou L (2010) Mental disorders and suicide among young rural Chinese: A case-control psychological autopsy study. American Journal of Psychiatry 167: 773-781.

35. Zhang J, Zhou L (2009) A case control study of suicides in China with and without mental disorder. Crisis 30: 68-72.

36. Phillips MR, Yang G, Zhang Y, Wang L, Ji H, et al. (2002) Risk factors for suicide in China: a national case-control psychological autopsy study Lancet 360 1728-1736.

37. Li XY, Phillips MR, Zhang YP, Xu D, Yang GH (2008) Risk factors for suicide in China’s youth: a case-control study. Psychological Medicine 38: 397-406.

38. Zhang J, Conwell Y, Zhou L, Jiang C (2004) Culture, risk factors and suicide in rural China: a psychological autopsy case control study Acta Psychiatrica Scandinavica 110: 430–437.

39. Cheng AT (1995) Mental illness and suicide. A case-control study in east Taiwan. Arch Gen Psychiatry 52: 594-603.

40. Vijayakumar L, Rajkumar S (1999) Are risk factors for suicide universal? A case-control study in India. Acta Psychiatrica Scandinavica 99: 407-411.

41. Gururaj G, Isaac MK, Subbakrishna DK, Ranjani R (2004) Risk factors for completed suicide: a case-control study from Bangalore, India International Journal of Injury Control and Safety Promotion 11.

42. Khan FA, Anand B, Devi MG, Murthy KK (2005) Psychological autopsy of suicide-a cross-sectional study. Indian J Psychiatry 47: 73-78.

43. Khan MM, Mahmud S, Karim MS, Zaman M, Prince M (2008) Case–control study of suicide in Karachi, Pakistan. British Journal of Psychiatry 193: 402-405.
