# Supplementary material for: The Burden Attributable to Mental and Substance Use Disorders as Risk Factors for Suicide: Findings from the Global Burden of Disease Study 2010
Source: PLoS One. 2014 Apr 2;9(4):e91936. doi: 10.1371/journal.pone.0091936 (PMC3973668; doi:10.1371/journal.pone.0091936)
Supplement: File S1 — This file contains Text S1 and Tables S1 to S6. (ZIP) [file pone.0091936.s001.zip › Supplemental files/Table S4_Ferrari et al_181013.docx]

### Table S4: Pooled proportion of suicide cases attributable to mental and substance use disorders

| **Region** | **Pooled proportion**  **(95% UI)** | **Number of studies** | **Number of countries** |
| --- | --- | --- | --- |
| **Group 1: China, India, Taiwan** |  |  |  |
| Overall | QE: 68.3% (55.2%-80.0%)  RE: 69.4% (53.5%-83.4%) | 9 | 3 |
| Male | QE: 63.4% (46.2%-79.1%)  RE: 76.3% (50.1%-90.6%) | 1 | 3 |
| Female | QE: 48.6% (29.1%-68.3%)  RE: 61.0% (30.5%-87.9%) | 1 | 3 |
| **Group 2: Other countries^a^** |  |  |  |
| Overall | QE: 84.5% (78.6%-89.6%)  RE: 83.8% (76.5%-90.0%) | 34 | 17 |
| Male | QE: 88.8% (84.1%-92.8%)  RE: 87.9% (82.9%-92.1%) | 12 | 7 |
| Female | QE: 93.3% (87.2%-97.6%)  RE: 94.2% (86.5%-99.0%) | 12 | 8 |

*Note.95% UI: 95% uncertainty interval; QE: Quality effect model estimate, RE: Random effects model estimate; ^a^Group 2 countries: Includes studies from Australia, USA, Canada, Colombia, Hungary, United Kingdom, Belgium, Italy, Germany, Sweden, Ireland, Norway, Finland, Indonesia, Pakistan;*
