# Supplementary material for: The Burden Attributable to Mental and Substance Use Disorders as Risk Factors for Suicide: Findings from the Global Burden of Disease Study 2010
Source: PLoS One. 2014 Apr 2;9(4):e91936. doi: 10.1371/journal.pone.0091936 (PMC3973668; doi:10.1371/journal.pone.0091936)
Supplement: File S1 — This file contains Text S1 and Tables S1 to S6. (ZIP) [file pone.0091936.s001.zip › Supplemental files/Table S5_Ferrari et al_181013.docx]

### Table S5: Suicide DALYs (i.e. YLLs) attributable to mental and substance use disorders in 1990 and 2010

|  | **1990** |  |  | **2010** |  |  |
| --- | --- | --- | --- | --- | --- | --- |
| **YLL** | **95% UI** | | **YLL** | **95% UI** | |  |
|  | *Mean* | *Lower* | *Upper* | *Mean* | *Lower* | *Upper* |
| **By sex** |  |  |  |  |  |  |
| **Male** | 11,400,000 | 7,700,000 | 15,800,000 | 14,900,000 | 95000,00 | 20,100,000 |
| **Female** | 6,800,000 | 4,000,000 | 9,900,000 | 7,600,000 | 4,400,000 | 10,600,000 |
| **By age** |  |  |  |  |  |  |
| **5-9 years** | 20,000 | 10,000 | 40,000 | 20,000 | 10,000 | 30,000 |
| **10-14 years** | 300,000 | 200,000 | 500,000 | 300,000 | 100,000 | 600,000 |
| **15-19 years** | 2,200,000 | 1,300,000 | 3,100,000 | 2,500,000 | 1,500,000 | 3,800,000 |
| **20-24 years** | 3,100,000 | 2,000,000 | 4,300,000 | 3,400,000 | 2,000,000 | 4,900,000 |
| **25-29 years** | 2,600,000 | 1,700,000 | 3,500,000 | 2,900,000 | 1,900,000 | 4,000,000 |
| **30-34 years** | 2,200,000 | 1,500,000 | 2,900,000 | 2,400,000 | 1,500,000 | 3,200,000 |
| **35-39 years** | 1,900,000 | 1,300,000 | 2,600,000 | 2,200,000 | 1,400,000 | 3,100,000 |
| **40-44 years** | 1,500,000 | 1,000,000 | 2,000,000 | 2,000,000 | 1,400,000 | 2,900,000 |
| **45-49 years** | 1,100,000 | 800,000 | 1,600,000 | 1,800,000 | 1,200,000 | 2,500,000 |
| **50-54 years** | 1,100,000 | 800,000 | 1,600,000 | 1,600,000 | 1,000,000 | 2,300,000 |
| **55-59 years** | 800,000 | 500,000 | 1,200,000 | 1,200,000 | 700,000 | 1,800,000 |
| **60-64 years** | 600,000 | 400,000 | 900,000 | 800,000 | 500,000 | 1,200,000 |
| **65-69 years** | 400,000 | 200,000 | 600,000 | 500,000 | 300,000 | 800,000 |
| **70-74 years** | 200,000 | 100,000 | 300,000 | 400,000 | 200,000 | 600,000 |
| **75-79 years** | 200,000 | 100,000 | 200,000 | 300,000 | 100,000 | 400,000 |
| **80 + years** | 100,000 | 100,000 | 200,000 | 200,000 | 100,000 | 300,000 |
| **By region** |  |  |  |  |  |  |
| **Asia Pacific, High Income** | 600,000 | 400,000 | 1,000,000 | 800,000 | 400,000 | 1,200,000 |
| **Asia, Central** | 300,000 | 200,000 | 400,000 | 400,000 | 200,000 | 500,000 |
| **Asia, East** | 5,300,000 | 2,900,000 | 7,400,000 | 3,500,000 | 2,200,000 | 6,100,000 |
| **Asia, South** | 4,000,000 | 2,600,000 | 6,000,000 | 8,800,000 | 4,800,000 | 12,600,000 |
| **Asia, Southeast** | 1,100,000 | 700,000 | 1,500,000 | 1,300,000 | 800,000 | 1,800,000 |

|  |  | **1990** |  |  | **2010** |  |
| --- | --- | --- | --- | --- | --- | --- |
|  | **YLL** | **95% UI** | | **YLL** | **95% UI** | |
|  | *Mean* | *Lower* | *Upper* | *Mean* | *Lower* | *Upper* |
| **By region** |  |  |  |  |  |  |
| **Australasia** | 80,000 | 50,000 | 100,000 | 70,000 | 50,000 | 100,000 |
| **Caribbean** | 100,000 | 100,000 | 100,000 | 100,000 | 100,000 | 100,000 |
| **Europe, Central** | 600,000 | 400,000 | 800,000 | 500,000 | 300,000 | 600,000 |
| **Europe, Eastern** | 2,000,000 | 1,400,000 | 2,700,000 | 1,800,000 | 1,200,000 | 2,800,000 |
| **Europe, Western** | 1,500,000 | 1,000,000 | 2,000,000 | 1,200,000 | 800,000 | 1,700,000 |
| **Latin America, Andean** | 50,000 | 40,000 | 70,000 | 90,000 | 50,000 | 100,000 |
| **Latin America, Central** | 200,000 | 160,000 | 400,000 | 400,000 | 200,000 | 500,000 |
| **Latin America, Southern** | 200,000 | 110,000 | 200,000 | 200,000 | 100,000 | 300,000 |
| **Latin America, Tropical** | 300,000 | 200,000 | 400,000 | 400,000 | 300,000 | 500,000 |
| **North Africa / Middle East** | 200,000 | 100,000 | 300,000 | 500,000 | 200,000 | 700,000 |
| **North America, High Income** | 1,100,000 | 700,000 | 1,400,000 | 1,100,000 | 800,000 | 1,500,000 |
| **Oceania** | 20,000 | 10,000 | 30,000 | 30,000 | 20,000 | 50,000 |
| **Sub-Saharan Africa, Central** | 80,000 | 50,000 | 100,000 | 200,000 | 100,000 | 300,000 |
| **Sub-Saharan Africa, East** | 400,000 | 300,000 | 600,000 | 800,000 | 500,000 | 1,100,000 |
| **Sub-Saharan Africa, Southern** | 100,000 | 50,000 | 200,000 | 200,000 | 100,000 | 300,000 |
| **Sub-Saharan Africa, West** | 100,000 | 60,000 | 200,000 | 200,000 | 100,000 | 300,000 |
| **By disorder** |  |  |  |  |  |  |
| **Alcohol dependence** | 4,200,000 | 3,200,000 | 5,300,000 | 4,900,000 | 3,600,000 | 6,300,000 |
| **Amphetamine dependence** | 700,000 | 300,000 | 1,400,000 | 900,000 | 300,000 | 1,700,000 |
| **Anorexia nervosa** | 40,000 | 10,000 | 100,000 | 60,000 | 10,000 | 200,000 |
| **Anxiety disorder** | 2,100,000 | 800,000 | 3,600,000 | 2,700,000 | 1,000,000 | 4,800,000 |
| **Bipolar disorder** | 1,600,000 | 500,000 | 3,300,000 | 2,000,000 | 600,000 | 4,000,000 |
| **Cocaine dependence** | 300,000 | 100,000 | 600,000 | 300,000 | 100,000 | 700,000 |
| **Major depressive disorder** | 13,500,000 | 8,000,000 | 18,900,000 | 16,700,000 | 9,900,000 | 23,300,000 |
| **Opioid dependence** | 500,000 | 300,000 | 800,000 | 700,000 | 400,000 | 1,100,000 |
| **Schizophrenia** | 1,400,000 | 1,000,000 | 1,700,000 | 1,700,000 | 1,300,000 | 2,200,000 |

*Note. DALYs: Disability adjusted life years; YLLs: years of life lost; 95% UI: 95% uncertainty interval; Absolute YLLs rounded to 100,000*
