# Supplementary material for: The Burden Attributable to Mental and Substance Use Disorders as Risk Factors for Suicide: Findings from the Global Burden of Disease Study 2010
Source: PLoS One. 2014 Apr 2;9(4):e91936. doi: 10.1371/journal.pone.0091936 (PMC3973668; doi:10.1371/journal.pone.0091936)
Supplement: File S1 — This file contains Text S1 and Tables S1 to S6. (ZIP) [file pone.0091936.s001.zip › Supplemental files/Table S6_Ferrari et al_181013.docx]

### Table S6: Suicide DALYs (i.e. YLLs) attributable to each mental and substance use disorders in 2010 by region

| **Region** | **Alcohol use disorder** | **Amphetamine dependence** | | **Anorexia nervosa** | **Anxiety disorder** | **Bipolar disorder** | **Cocaine dependence** | **Major depressive disorder** | **Opio**i**d dependence** | **Schizophrenia** |
| --- | --- | --- | --- | --- | --- | --- | --- | --- | --- | --- |
| **Asia Pacific, High Income** | |  |  | |  |  |  |  |  |  |
| *Mean* | 190,000 | 30,000 | | 10,000 | 90,000 | 70,000 | 20,000 | 520,000 | 30,000 | 70,000 |
| *95% UI: Lower* | 250,000 | 70,000 | | 40,000 | 170,000 | 160,000 | 50,000 | 860,000 | 70,000 | 90,000 |
| *Upper* | 110,000 | 10,000 | | 2,000 | 30,000 | 20,000 | 10,000 | 240,000 | 10,000 | 40,000 |
| **Asia, Central** |  |  | |  |  |  |  |  |  |  |
| *Mean* | 120,000 | 20,000 | | 300 | 50,000 | 30,000 | 4,000 | 240,000 | 10,000 | 30,000 |
| *95% UI: Lower* | 180,000 | 30,000 | | 1,000 | 90,000 | 60,000 | 10,000 | 370,000 | 20,000 | 50,000 |
| *Upper* | 90,000 | 10,000 | | 50 | 20,000 | 10,000 | 2,000 | 140,000 | 10,000 | 20,000 |
| **Asia, East** |  |  | |  |  |  |  |  |  |  |
| *Mean* | 870,000 | 100,000 | | 4,000 | 260,000 | 330,000 | 10,000 | 2,480,000 | 70,000 | 340,000 |
| *95% UI: Lower* | 1,450,000 | 220,000 | | 10,000 | 520,000 | 700,000 | 20,000 | 4,450,000 | 140,000 | 560,000 |
| *Upper* | 590,000 | 30,000 | | 1,000 | 90,000 | 100,000 | 3,000 | 1,250,000 | 30,000 | 240,000 |
| **Asia, South** |  |  | |  |  |  |  |  |  |  |
| *Mean* | 1,820,000 | 370,000 | | 2,000 | 1,090,000 | 770,000 | 100,000 | 6,530,000 | 310,000 | 600,000 |
| *95% UI: Lower* | 2,630,000 | 800,000 | | 10,000 | 2,020,000 | 1,620,000 | 220,000 | 9,730,000 | 530,000 | 820,000 |
| *Upper* | 1,020,000 | 110,000 | | 300 | 410,000 | 250,000 | 30,000 | 3,280,000 | 150,000 | 330,000 |
| **Asia, Southeast** |  |  | |  |  |  |  |  |  |  |
| *Mean* | 210,000 | 90,000 | | 1,000 | 140,000 | 120,000 | 4,000 | 970,000 | 30,000 | 100,000 |
| *95% UI: Lower* | 300,000 | 180,000 | | 2,000 | 270,000 | 250,000 | 10,000 | 1,430,000 | 50,000 | 150,000 |
| *Upper* | 160,000 | 30,000 | | 100 | 50,000 | 40,000 | 1,000 | 570,000 | 10,000 | 70,000 |
| **Australasia** |  |  | |  |  |  |  |  |  |  |
| *Mean* | 20,000 | 5,000 | | 1,000 | 10,000 | 10,000 | 2,000 | 50,000 | 4,000 | 10,000 |
| *95% UI: Lower* | 20,000 | 10,000 | | 2,000 | 20,000 | 10,000 | 4,000 | 70,000 | 10,000 | 10,000 |
| *Upper* | 10,000 | 2,000 | | 100 | 5,000 | 2,000 | 1,000 | 30,000 | 2,000 | 5,000 |
| **Caribbean** |  |  | |  |  |  |  |  |  |  |
| *Mean* | 20,000 | 3,000 | | 200 | 10,000 | 10,000 | 5,000 | 70,000 | 3,000 | 10,000 |
| *95% UI: Lower* | 30,000 | 10,000 | | 1,000 | 20,000 | 20,000 | 10,000 | 90,000 | 10,000 | 10,000 |
| *Upper* | 20,000 | 1,000 | | 30 | 4,000 | 2,000 | 2,000 | 40,000 | 1,000 | 5,000 |

| **Region** | **Alcohol use disorder** | **Amphetamine dependence** | **Anorexia nervosa** | **Anxiety disorder** | **Bipolar disorder** | **Cocaine dependence** | **Major depressive disorder** | **Opio**i**d dependence** | **Schizophrenia** |
| --- | --- | --- | --- | --- | --- | --- | --- | --- | --- |
| **Europe, Central** |  |  |  |  |  |  |  |  |  |
| *Mean* | 110,000 | 20,000 | 1,000 | 70,000 | 40,000 | 4,000 | 340,000 | 10,000 | 50,000 |
| *95% UI: Lower* | 150,000 | 50,000 | 3,000 | 130,000 | 90,000 | 10,000 | 490,000 | 20,000 | 70,000 |
| *Upper* | 80,000 | 10,000 | 200 | 30,000 | 10,000 | 1,000 | 200,000 | 10,000 | 30,000 |
| **Europe, Eastern** |  |  |  |  |  |  |  |  |  |
| *Mean* | 540,000 | 30,000 | 2,000 | 160,000 | 150,000 | 10,000 | 1,410,000 | 50,000 | 130,000 |
| *95% UI: Lower* | 850,000 | 80,000 | 10,000 | 360,000 | 320,000 | 30,000 | 2,240,000 | 110,000 | 210,000 |
| *Upper* | 400,000 | 10,000 | 300 | 50,000 | 50,000 | 5,000 | 820,000 | 30,000 | 90,000 |
| **Europe, Western** |  |  |  |  |  |  |  |  |  |
| *Mean* | 280,000 | 40,000 | 10,000 | 170,000 | 90,000 | 20,000 | 920,000 | 40,000 | 70,000 |
| *95% UI: Lower* | 380,000 | 80,000 | 40,000 | 290,000 | 190,000 | 50,000 | 1,330,000 | 70,000 | 90,000 |
| *Upper* | 220,000 | 10,000 | 2,000 | 70,000 | 30,000 | 10,000 | 550,000 | 20,000 | 50,000 |
| **Latin America, Andean** |  |  |  |  |  |  |  |  |  |
| *Mean* | 30,000 | 3,000 | 100 | 10,000 | 10,000 | 4,000 | 60,000 | 3,000 | 10,000 |
| *95% UI: Lower* | 40,000 | 10,000 | 200 | 30,000 | 10,000 | 10,000 | 90,000 | 10,000 | 10,000 |
| *Upper* | 20,000 | 1,000 | 10 | 4,000 | 2,000 | 1,000 | 30,000 | 1,000 | 3,000 |
| **Latin America, Central** |  |  |  |  |  |  |  |  |  |
| *Mean* | 80,000 | 20,000 | 1,000 | 60,000 | 40,000 | 10,000 | 300,000 | 10,000 | 30,000 |
| *95% UI: Lower* | 100,000 | 50,000 | 2,000 | 100,000 | 80,000 | 20,000 | 430,000 | 20,000 | 40,000 |
| *Upper* | 60,000 | 10,000 | 100 | 20,000 | 10,000 | 3,000 | 160,000 | 10,000 | 20,000 |
| **Latin America, Southern** |  |  |  |  |  |  |  |  |  |
| *Mean* | 40,000 | 10,000 | 1,000 | 30,000 | 20,000 | 10,000 | 150,000 | 10,000 | 10,000 |
| *95% UI: Lower* | 60,000 | 20,000 | 2,000 | 60,000 | 40,000 | 20,000 | 210,000 | 10,000 | 20,000 |
| *Upper* | 30,000 | 3,000 | 100 | 10,000 | 10,000 | 3,000 | 90,000 | 3,000 | 10,000 |
| **Latin America, Tropical** |  |  |  |  |  |  |  |  |  |
| *Mean* | 80,000 | 20,000 | 200 | 60,000 | 30,000 | 20,000 | 310,000 | 10,000 | 30,000 |
| *95% UI: Lower* | 120,000 | 40,000 | 1,000 | 100,000 | 70,000 | 40,000 | 430,000 | 20,000 | 50,000 |
| *Upper* | 50,000 | 10,000 | 30 | 20,000 | 10,000 | 10,000 | 190,000 | 4,000 | 10,000 |

| **Region** | **Alcohol use disorder** | **Amphetamine dependence** | | **Anorexia nervosa** | **Anxiety disorder** | **Bipolar disorder** | **Cocaine dependence** | **Major depressive disorder** | **Opio**i**d dependence** | **Schizophrenia** |
| --- | --- | --- | --- | --- | --- | --- | --- | --- | --- | --- |
| **North Africa / Middle East** |  |  | |  |  |  |  |  |  |  |
| *Mean* | 40,000 | 20,000 | | 2,000 | 80,000 | 40,000 | 10,000 | 400,000 | 20,000 | 40,000 |
| *95% UI: Lower* | 50,000 | 50,000 | | 10,000 | 160,000 | 90,000 | 30,000 | 580,000 | 40,000 | 50,000 |
| *Upper* | 20,000 | 10,000 | | 300 | 30,000 | 10,000 | 4,000 | 190,000 | 10,000 | 20,000 |
| **North America, High Income** | |  |  | |  |  |  |  |  |  |
| *Mean* | 220,000 | 30,000 | | 10,000 | 200,000 | 100,000 | 60,000 | 850,000 | 30,000 | 120,000 |
| *95% UI: Lower* | 270,000 | 70,000 | | 40,000 | 350,000 | 200,000 | 130,000 | 1,180,000 | 50,000 | 150,000 |
| *Upper* | 160,000 | 10,000 | | 2,000 | 80,000 | 30,000 | 20,000 | 510,000 | 20,000 | 80,000 |
| **Oceania** |  |  | |  |  |  |  |  |  |  |
| *Mean* | 10,000 | 1,000 | | 10 | 4,000 | 3,000 | 200 | 20,000 | 1,000 | 2,000 |
| *95% UI: Lower* | 10,000 | 3,000 | | 40 | 10,000 | 10,000 | 400 | 40,000 | 2,000 | 10,000 |
| *Upper* | 4,000 | 300 | | 2 | 1,000 | 1,000 | 40 | 10,000 | 300 | 1,000 |
| **Sub-Saharan Africa, Central** | |  |  | |  |  |  |  |  |  |
| *Mean* | 20,000 | 10,000 | | 1,000 | 30,000 | 20,000 | 1,000 | 140,000 | 3,000 | 10,000 |
| *95% UI: Lower* | 40,000 | 20,000 | | 2,000 | 60,000 | 30,000 | 3,000 | 220,000 | 10,000 | 20,000 |
| *Upper* | 10,000 | 2,000 | | 100 | 10,000 | 4,000 | 400 | 80,000 | 1,000 | 10,000 |
| **Sub-Saharan Africa, East** |  |  | |  |  |  |  |  |  |  |
| *Mean* | 110,000 | 40,000 | | 100 | 120,000 | 70,000 | 10,000 | 650,000 | 20,000 | 50,000 |
| *95% UI: Lower* | 150,000 | 80,000 | | 200 | 220,000 | 140,000 | 10,000 | 930,000 | 30,000 | 60,000 |
| *Upper* | 70,000 | 10,000 | | 10 | 50,000 | 20,000 | 2,000 | 380,000 | 10,000 | 30,000 |
| **Sub-Saharan Africa, Southern** | |  |  | |  |  |  |  |  |  |
| *Mean* | 60,000 | 10,000 | | 100 | 20,000 | 20,000 | 2,000 | 160,000 | 10,000 | 10,000 |
| *95% UI: Lower* | 90,000 | 20,000 | | 300 | 50,000 | 40,000 | 5,000 | 240,000 | 10,000 | 20,000 |
| *Upper* | 40,000 | 4,000 | | 10 | 10,000 | 5,000 | 1,000 | 80,000 | 2,000 | 10,000 |
| **Sub-Saharan Africa, West** |  |  | |  |  |  |  |  |  |  |
| *Mean* | 20,000 | 10,000 | | 10 | 20,000 | 20,000 | 2,000 | 140,000 | 4,000 | 10,000 |
| *95% UI: Lower* | 20,000 | 20,000 | | 40 | 50,000 | 40,000 | 4,000 | 220,000 | 10,000 | 20,000 |
| *Upper* | 10,000 | 3,000 | | 2 | 10,000 | 5,000 | 1,000 | 80,000 | 2,000 | 10,000 |

*Note. DALYs: Disability adjusted life years; YLLs: years of life lost; 95% UI: 95% uncertainty interval; Absolute YLLs rounded to 100,000*
