# Supplementary material for: The Burden Attributable to Mental and Substance Use Disorders as Risk Factors for Suicide: Findings from the Global Burden of Disease Study 2010
Source: PLoS One. 2014 Apr 2;9(4):e91936. doi: 10.1371/journal.pone.0091936 (PMC3973668; doi:10.1371/journal.pone.0091936)
Supplement: File S1 — This file contains Text S1 and Tables S1 to S6. (ZIP) [file pone.0091936.s001.zip › Supplemental files/Text S1_Ferrari et al_181013.docx]

**Supporting Text S1**

**PRISMA checklist and flow diagram for the literature search to identify relative-risk estimates**

We used data sources from recent and methodologically comparable systematic reviews of the association between suicide and mental and substance use disorders [[1-5](#_ENREF_1)], specifically affective disorders, anxiety disorders, schizophrenia [[3](#_ENREF_3)], cocaine, opioid, and amphetamine dependence [[1](#_ENREF_1),[2](#_ENREF_2),[4](#_ENREF_4)] and alcohol dependence [[5](#_ENREF_5)] . We expanded the Li and collaborators systematic review and replicated the literature search [[3](#_ENREF_3)] to collect data for bipolar disorder and MDD separately (rather than affective disorders combined), and anorexia nervosa which was not included in the original review.

The PRISMA checklist and flowchart [[6](#_ENREF_6)], for this literature search have been summarised below. The information presented amalgamate the search for data previously reported[[1-5](#_ENREF_1)], as well as the expansion of the Li and collaborators systematic review [[3](#_ENREF_3)] to collect data for bipolar disorder and MDD separately.

| **Section/topic** | **#** | **Checklist item** | **Reported on page #** |
| --- | --- | --- | --- |
| **TITLE** | | |  |
| Title | 1 | Identify the report as a systematic review, meta-analysis, or both. | N/A. This was reported in the specific review papers [[1-5](#_ENREF_1)]. |
| **ABSTRACT** | | |  |
| Structured summary | 2 | Provide a structured summary including, as applicable: background; objectives; data sources; study eligibility criteria, participants, and interventions; study appraisal and synthesis methods; results; limitations; conclusions and implications of key findings; systematic review registration number. | Page 2 |
| **INTRODUCTION** | | |  |
| Rationale | 3 | Describe the rationale for the review in the context of what is already known. | Pages 3-4 |
| Objectives | 4 | Provide an explicit statement of questions being addressed with reference to participants, interventions, comparisons, outcomes, and study design (PICOS). | Pages 3-4 |
| **METHODS** | | |  |
| Protocol and registration | 5 | Indicate if a review protocol exists, if and where it can be accessed (e.g., Web address), and, if available, provide registration information including registration number. | N/A |
| Eligibility criteria | 6 | Specify study characteristics (e.g., PICOS, length of follow-up) and report characteristics (e.g., years considered, language, publication status) used as criteria for eligibility, giving rationale. | Summary provided on pages 5-6 with more details in specific review papers [[1-5](#_ENREF_1)]. |
| Information sources | 7 | Describe all information sources (e.g., databases with dates of coverage, contact with study authors to identify additional studies) in the search and date last searched. | Summary provided on page 6 with more details in Text S1 and the specific review papers [[1-5](#_ENREF_1)]. |
| Search | 8 | Present full electronic search strategy for at least one database, including any limits used, such that it could be repeated. | Summary provided on page 6 with more details in the specific review papers [[1-5](#_ENREF_1)]. |
| Study selection | 9 | State the process for selecting studies (i.e., screening, eligibility, included in systematic review, and, if applicable, included in the meta-analysis). | Pages 5-6 |
| Data collection process | 10 | Describe method of data extraction from reports (e.g., piloted forms, independently, in duplicate) and any processes for obtaining and confirming data from investigators. | Summary provided on page 6 with more details in Table S1 and the specific review papers [[1-5](#_ENREF_1)]. |
| Data items | 11 | List and define all variables for which data were sought (e.g., PICOS, funding sources) and any assumptions and simplifications made. | Summary provided on pages 5-6 with more details in Table S1. |
| Risk of bias in individual studies | 12 | Describe methods used for assessing risk of bias of individual studies (including specification of whether this was done at the study or outcome level), and how this information is to be used in any data synthesis. | Strategies for adjusting study- and country-level sources of variability through (1) the quality-effects model and (2) ceiling values for joint population attributable fractions discussed on pages 6-8 with more details in Tables S1, S2, S3, S4 |
| Summary measures | 13 | State the principal summary measures (e.g., risk ratio, difference in means). | Page 5 |
| Synthesis of results | 14 | Describe the methods of handling data and combining results of studies, if done, including measures of consistency (e.g., I^2^) for each meta-analysis. | Pages 5, 6, 9 |

| **Section/topic** | **#** | **Checklist item** | **Reported on page #** |
| --- | --- | --- | --- |
| Risk of bias across studies | 15 | Specify any assessment of risk of bias that may affect the cumulative evidence (e.g., publication bias, selective reporting within studies). | Strategies for adjusting study- and country-level sources of variability through (1) the quality-effects model and (2) ceiling values for joint population attributable fractions discussed on pages 6-8 with more details in Tables S1, S2, S3, S4. More strategies for addressing publication bias and selective reporting were presented in the specific review papers [[1-5](#_ENREF_1)] |
| Additional analyses | 16 | Describe methods of additional analyses (e.g., sensitivity or subgroup analyses, meta-regression), if done, indicating which were pre-specified. | Pages 6-7 discuss the sex-, region, and disorder-specific analyses conducted. As-well as sensitivity analyses around the type of model used to pool estimates in the meta-analysis. |
| **RESULTS** | | |  |
| Study selection | 17 | Give numbers of studies screened, assessed for eligibility, and included in the review, with reasons for exclusions at each stage, ideally with a flow diagram. | Summary provided on pages 9-10, Table S1, with a literature search flow diagram in Text S1. |
| Study characteristics | 18 | For each study, present characteristics for which data were extracted (e.g., study size, PICOS, follow-up period) and provide the citations. | Summary provided on pages 9-10 with more detail in Table S1. |
| Risk of bias within studies | 19 | Present data on risk of bias of each study and, if available, any outcome level assessment (see item 12). | Pages 9-10, Tables S2 and S4 compare findings of the meta-analysis conducted using random- and quality-effects models. |
| Results of individual studies | 20 | For all outcomes considered (benefits or harms), present, for each study: (a) simple summary data for each intervention group (b) effect estimates and confidence intervals, ideally with a forest plot. | Pages 9-10, and Tables 1, S1, S2, S3, S4 |
| Synthesis of results | 21 | Present results of each meta-analysis done, including confidence intervals and measures of consistency. | Pages 9-10, and Tables 1, S1, S2, S3, S4 |
| Risk of bias across studies | 22 | Present results of any assessment of risk of bias across studies (see Item 15). | Assessments for adjusting study- and country-level sources of variability through (1) the quality-effects model and (2) ceiling values for joint population attributable fractions presented in Tables S1, S2, S3, S4. More strategies for addressing publication bias and selective reporting were presented in specific review papers [[1-5](#_ENREF_1)] |
| Additional analysis | 23 | Give results of additional analyses, if done (e.g., sensitivity or subgroup analyses, meta-regression [see Item 16]). | Assessments of findings by sex-, region, and disorder presented in Pages 9-10, Tables 1, S2, S4 |
| **DISCUSSION** | | |  |
| Summary of evidence | 24 | Summarize the main findings including the strength of evidence for each main outcome; consider their relevance to key groups (e.g., healthcare providers, users, and policy makers). | Pages 12-15 |
| Limitations | 25 | Discuss limitations at study and outcome level (e.g., risk of bias), and at review-level (e.g., incomplete retrieval of identified research, reporting bias). | Pages 14-15 |
| Conclusions | 26 | Provide a general interpretation of the results in the context of other evidence, and implications for future research. | Page 12-14 |

| **FUNDING** | | |  |
| --- | --- | --- | --- |
| Funding | 27 | Describe sources of funding for the systematic review and other support (e.g., supply of data); role of funders for the systematic review. | Funding information providing in the ‘Additional Information’ section of PloS One’s online submission form. |

*From:*  Moher D, Liberati A, Tetzlaff J, Altman DG, The PRISMA Group (2009). Preferred Reporting Items for Systematic Reviews and Meta-Analyses: The PRISMA Statement. PLoS Med 6(6): e1000097. doi:10.1371/journal.pmed1000097

For more information, visit: **www.prisma-statement.org**.


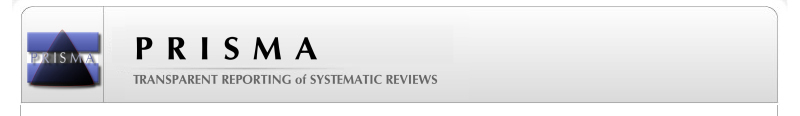
**PRISMA 2009 Flow Diagram**

Studies included in qualitative synthesis
(n = 55)

Full-text articles assessed for eligibility
(n = 485)

Records excluded
(n = 7504)

Reaso

Records screened
(n =7989)

Records after duplicates removed
(n =7989)

Additional records identified through other sources
(n = 117)

## Identification

## Eligibility

## Included

## Screening

Records identified through database searching
(n = 7872)

Full-text articles excluded,
(n = 430)

Main reasons for exclusion;

. Only secondary data cited.

. Incomplete data to assess methodological quality

. Effect-size not reported or insufficient data to estimate effect-size.

. Sampling strategy or study type not representative.

. Clinical diagnostic criteria not used.

Studies included in quantitative synthesis for mental disorders
(n = depression: 4; anxiety disorder: 7; bipolar disorder: 4; schizophrenia: 4; anorexia nervosa: 9)

Studies included in quantitative synthesis for illicit drug use dependence
(n = opioid: 21; cocaine: 3; amphetamine: 1)

Studies included in quantitative synthesis for alcohol dependence
(n = 12)

**References**

1. Degenhardt L, Bucello C, Mathers B, Briegleb C, Ali H, et al. (2011) Mortality among problematic users of heroin and other illicit opioids: A systematic review and meta-analysis. Addiction 106: 32-51.

2. Degenhardt L, Singleton J, Calabria B, McLaren J, Kerr T, et al. (2011) Mortality among cocaine users: A systematic review of cohort studies. Drug and Alcohol Dependence 113: 88-95.

3. Li Z, Page A, Martin G, Taylor R (2011) Attributable risk of psychiatric and socio-economic factors for suicide from individual-level, population-based studies: a systematic review. Soc Sci Med 72: 608-616.

4. Singleton J, Degenhardt L, Hall W, Zabransky T (2009) Mortality among people who use amphetamines: A systematic review of cohort studies. Drug & Alcohol Dependence 105: 1-8.

5. Wilcox HC, Conner KR, Caine ED (2004) Association of alcohol and drug use disorders and completed suicide: an empirical review of cohort studies. Drug Alcohol Depend 76 Suppl: S11-19.

6. Moher D, Liberati A, Tetzlaff J, Altman DG, The PG (2009) Preferred reporting items for systematic reviews and meta-analyses: the PRISMA Statement. PLoS Med 6: e1000097.
